# Evaluation of the Distribution and Impacts of Parasites, Pathogens, and Pesticides on Honey Bee (*Apis mellifera*) Populations in East Africa

**DOI:** 10.1371/journal.pone.0094459

**Published:** 2014-04-16

**Authors:** Elliud Muli, Harland Patch, Maryann Frazier, James Frazier, Baldwyn Torto, Tracey Baumgarten, Joseph Kilonzo, James Ng'ang'a Kimani, Fiona Mumoki, Daniel Masiga, James Tumlinson, Christina Grozinger

**Affiliations:** 1 The International Centre of Insect Physiology and Ecology (icipe), Nairobi, Kenya; 2 Department of Biological Sciences, South Eastern Kenya University (SEKU), Kitui, Kenya; 3 Department of Entomology, Center for Pollinator Research, Pennsylvania State University, University Park, Pennsylvania, United States of America; The University of Melbourne, Australia

## Abstract

In East Africa, honey bees (*Apis mellifera*) provide critical pollination services and income for small-holder farmers and rural families. While honey bee populations in North America and Europe are in decline, little is known about the status of honey bee populations in Africa. We initiated a nationwide survey encompassing 24 locations across Kenya in 2010 to evaluate the numbers and sizes of honey bee colonies, assess the presence of parasites (*Varroa* mites and *Nosema* microsporidia) and viruses, identify and quantify pesticide contaminants in hives, and assay for levels of hygienic behavior. *Varroa* mites were present throughout Kenya, except in the remote north. Levels of *Varroa* were positively correlated with elevation, suggesting that environmental factors may play a role in honey bee host-parasite interactions. Levels of *Varroa* were negatively correlated with levels of hygienic behavior: however, while *Varroa* infestation dramatically reduces honey bee colony survival in the US and Europe, in Kenya *Varroa* presence alone does not appear to impact colony size. *Nosema apis* was found at three sites along the coast and one interior site. Only a small number of pesticides at low concentrations were found. Of the seven common US/European honey bee viruses, only three were identified but, like *Varroa*, were absent from northern Kenya. The number of viruses present was positively correlated with *Varroa* levels, but was not correlated with colony size or hygienic behavior. Our results suggest that *Varroa*, the three viruses, and *Nosema* have been relatively recently introduced into Kenya, but these factors do not yet appear to be impacting Kenyan bee populations. Thus chemical control for *Varroa* and *Nosema* are not necessary for Kenyan bees at this time. This study provides baseline data for future analyses of the possible mechanisms underlying resistance to and the long-term impacts of these factors on African bee populations.

## Introduction

Pollinators are essential contributors to global nutrition and food security. An estimated three-quarters of major global food crops benefit from pollinators [1]. Fruits, vegetables, and nuts, which provide key vitamins, minerals, fats and other micronutrients are particularly dependent on pollinators [2], and thus pollinators form a crucial line of defense against micronutrient deficiencies in developing countries. Furthermore, the productivity of many high value crops grown in the developing world, such as cacao, coffee, and cashews, is strongly tied to pollination services [3]–[6]. Indeed, the amount of animal pollinated crops grown globally has increased significantly in the last fifty years [7], making both developed- and developing world countries increasingly dependent on pollinator populations for food security and production of economically important crops.

Globally, pollination services amount to $212 billion, corresponding to ∼9.5% of the total value of world agriculture production for human consumption in 2005 [3]. Honey bees (*Apis mellifera*) are one of the most important pollinators worldwide, contributing $14.6 billion in pollination services to the US in 2000 [8] and $3.2 billion to the South African economy in 1998 [9]. However, honey bee populations have been in decline in North America and Europe over the last ∼30 years, with beekeepers routinely losing 30% of their managed colonies every winter during the last 7 years [10]. Several factors have been shown to negatively impact the longevity of honey bee colonies, including parasites (primarily *Varroa* mites [11] and *Nosema* microsporidia [12]), pathogens (22 different viruses have been identified [13], [14], along with several bacterial and fungal brood pathogens [15], [16]), pesticide exposure [17], poor nutrition [18], reduced genetic diversity [19], and management practices [20]. Large-scale surveys of managed honey bee populations in the US and Europe have failed to identify a single factor that is consistently strongly correlated with colony losses, leading researchers to believe a combination of factors acts synergistically to reduce survival [21]–[25].

In East Africa, honey bees provide critical pollination services, nutrition, and income for small-holder farmers and rural families. There is considerable genetic diversity in *Apis mellifera* populations in this region: indeed, five distinct *Apis mellifera* subspecies, each adapted to a specific ecological niche, have been identified in Kenya and in the surrounding region [26]–[29]. These bee populations are unmanaged: typically beekeepers set out empty receptacles (traditionally, hollowed-out logs), and bee swarms will occupy them as they migrate into the area [30], [31]. In western Kenya, pollinators provide USD $3.2 million in ecosystems services to 8 crops (beans, cowpeas, butternuts, sunflower, monkeynut, tomatoes, capsicum and passion fruit, [32]). Furthermore, the honey collected from these colonies serves as an important source of nutrition and income for families. Currently, Kenya is a net importer of honey (over 10 metric tons in 2005, [33]), and thus honey and potentially beeswax production could be improved upon as a viable source of income for many rural communities.

Discussions with beekeepers in 2010, data from the Kenyan National Beekeeping Station, and the personal experience and observations of the Kenyan authors indicate that over the past five to seven years there has been a significant decline in the number of hives that are being colonized, reduction in the size of migratory swarms, and decrease in honey production [34]. Beekeepers noted that in the past, empty hives were colonized in a matter of weeks during the swarming season, whereas now it could take months and many hives remain uncolonized. Recently beekeepers complained about fist-size swarms that they chase out of hives knowing these will not result in productive colonies. According to nation-wide data collected by the National Beekeeping Station in 2005, hives in Kenya numbered 1,356,534 and average honey production was 20.28 kg/hive. In 2006 hives numbered 1,241,604 but honey production dropped to 15 kg/hive and in 2007, hive numbers increased to 1,575,978 but honey production dropped to 9.3 kg/colony [34]. This reduced performance may be disease related because in 2009, we identified *Varroa* mites in honey bee colonies in East Africa for the first time [35]. A previous survey conducted between 1996 and 1998 ([36]; Shi Wei, personal communication, 2011) did not detect *Varroa* and it thus appears to be a relatively recent introduction. The introduction of *Varroa* mites to South Africa in 1997 was associated with large losses of managed honey bee colonies [9]. Furthermore, *Varroa* mites have been shown to vector several honey bee viruses [37], [38], which can also negatively impact honey bee health [13].

**Figure 1:**
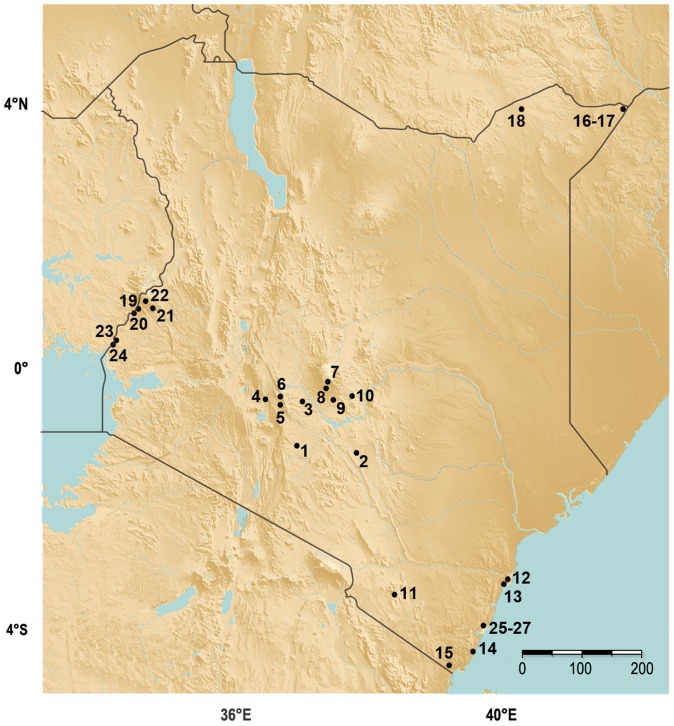
Geographic location of surveyed apiaries. Twenty-four apiaries were surveyed throughout Kenya with an additional three apiaries (25–27), see supplemenatry material, surveyed for ecological effects on colony health. The location and numerical designation of the apiaries is indicated on the map.

In 2010, we initiated a nationwide survey to obtain comprehensive information about the distribution of parasites, pathogens and pesticides in honey bee populations throughout Kenya, and determine if these are correlated with honey bee health, as measured in terms of colony size. In 24 locations across the country we assessed the numbers and sizes of honey bee colonies, the presence of *Varroa*, *Nosema*, and viruses, identified and quantified pesticide contaminants in hives, and assayed for levels of hygienic behavior (removal of dead pupae by adult bees, which is a measure of resistance to brood diseases and parasites such as *Varroa*) in colonies (see Figure 1 for apiary locations). We further analyzed this data set to determine if there were associations between *Varroa* loads, viral diversity, hygienic behavior, subspecies, location (which is correlated with elevation), and colony size. Given the relatively recent introduction of *Varroa* into this region, the large degree of genetic and ecological diversity, and the lack of confounding factors introduced by intensive management practices, this survey provides an unprecedented opportunity to examine the factors affecting honey bee health, lays the groundwork for long-term monitoring of bee populations in this region, and provides important information for the conservation of populations of this key pollinator species in East Africa.

## Materials and Methods

### General survey information

We surveyed and collected samples from 24 apiaries, comprising 81 colonies total, across Kenya (Figure 1). Compiled detailed information and results related to each apiary and colony are available in Table S1 and summaries of the types of data obtained from each apiary are provided in Table S2. All samples were collected at maintained apiaries and consent was given by the collaborating authority. In all cases the owner, in the case of private land, or relevant authority, in the case of public land, gave permission for collections. See Form S1 for a list of the apiaries and owners. Apiaries 1–15 were surveyed in June 2010, and apiaries 16–24 were surveyed in July - September 2010. For each colony, foragers returning to the hive entrance with pollen were collected in RNAlater (Qiagen, Valencia, CA) or 95% ethanol. Abdomens of the RNAlater-stored bees were pierced with sterile scalpel blades to expose soft tissues to the RNAlater preservative. These samples were used for viral detection. Bees collected in EtOH were used for *Nosema* detection and for subspecies identification. Bees were collected into individual 2 ml cryogenic vials (VWR, Radnor, PA). Samples were collected on ice, stored at −20°C during field collections, and then shipped to Penn State University (University Park, PA) within a month. At Penn State, RNAlater samples were stored at −80°C and all EtOH samples were stored at 4°C. When brood nests were accessible (for example, the interiors of colonies housed in traditional log hives could not be sampled without destroying the colony) wax and stored pollen samples were collected into sterile Whirl-Pak bags (Nasco, Fort Atkinson, WI) for pesticide analysis. When possible, colonies and apiaries were surveyed for population health (percentage of occupied hives in an apiary), colony size (number of frames of adult and immature bees, hygienic behavior (removal of dead pupae), and numbers of *Varroa* mites. Since it was not possible to obtain information on all parameters from each colony/apiary in the survey, it was necessary to use different subsets of colonies/apiaries to examine and statistically analyze correlations among these parameters. Details of the specific assays, collections, and associated statistical analyses are below.

### Survey of *Varroa* mites

The presence and quantity of *Varroa* mites were assessed using a standard sugar roll assay described in [39], using a half-cup measuring cup to collect approximately 350 bees. This assay was performed on colonies from 19 apiaries; apiaries 2, 16, 19, 22 and 24 were not assessed.

Colonies at twelve apiaries (sites 1, 3, 4, 10, 11, 12, 13, 14, 15, 20, 21, 23) were used to examine the correlation between *Varroa* presence and elevation. These apiaries were selected because *Varroa* had been sampled and was present (note that *Varroa* was absent from apiaries 17 and 18, but this was likely because these regions are geographically quite distant and isolated and *Varroa* may not yet have been introduced), and the majority had five sampled colonies/apiary (sites 10, 14 and 15 had 3, 2, and 4 colonies, respectively). In order to obtain a normal distribution of the data, *Varroa* counts were converted to logarithmic scale using the following equation: log number of *Varroa*  = log_10_ (*Varroa* count +1). All subsequent statistical analyses for *Varroa* counts were performed using logarithmic scale. The log number of *Varroa* in each colony was correlated with elevation using a correlation analysis in JMP 9.0.2 (SAS, Cary, NC).

Eleven apiaries (sites 1, 3, 4, 10, 11, 12, 13, 14, 15, 20, 21) were used to examine the correlation between *Varroa* presence and colony size. Colony size was measured as the number of frames of bees. The log number of *Varroa*/colony was correlated with the number of frames of bees/colony using a correlation analysis in JMP 9.0.2 (SAS, Cary, NC).

### Survey of *Nosema*

Sixteen apiaries (sites 1, 3, 4, 10, 11, 12, 13, 14, 15, 16, 17, 18, 20, 21, 22, 23) were screened for the presence of *Nosema* microsporidia. DNA was extracted from pools of 5 foragers/colony (collected in 95% ethanol) using a CTAB buffer (100 mM Tris HCl, pH 8.0; 20 mM EDTA, pH 8.0; 1.4 M NaCl; 2% (w/v) cetyltrimethylammonium bromide; 0.2% (v/v) 2-mercaptoethanol) plus proteinase K overnight incubation at 55°C followed by a phenol/chloroform/isoamyl alcohol (25∶24∶1) extraction. The species of *Nosema* present in the samples was confirmed using a PCR-RFLP of partial small subunit (SSU) rRNA gene as in [40], see Table 1 for primer sequences. The SSU fragment was amplified in a 25 µl PCR reaction containing 1×PCR Buffer, 2.5 mM MgCL_2_, 200 µM of each dNTP, 0.5 µM of forward and reverse primer, 2.5 units of platinum Taq polymerase (Invitrogen, Grand Island, NY), and 1000 ng of template DNA. The PCR conditions were as follows: 95°C for 4 minutes, followed by 45 cycles of 95°C (60 s), 48°C (60 s), and 72°C (60 s), and a final extension step at 72°C for 4 minutes. The PCR products were separated on a 1% agarose gel and visualized with ethidium bromide. The 400 bp PCR amplicon was subjected to two double digest RFLP reactions with the restriction enzymes MspI and either NdeI or PacI (New England Biolabs, Ipswich, MA) at 37°C for 3 hours. The resulting fragments were separated on a 2% agarose gel and visualized with ethidium bromide to determine their sizes. Furthermore, the 400 bp fragments were also gel purified and extracted with the Qiaquick gel extraction kit (Qiagen, Valencia, CA) and sequenced at the Penn State Genomics Core Facility (University Park, PA). In order to increase our ability to detect *Nosema* infections, we monitored infections in foragers (levels and prevalence of *Nosema* are highest in foragers) and used a molecular approach, which is not only more sensitive than screening for spores using light microscopy, but can also detect the vegetative forms of *Nosema* [41], [65]. While several studies have successfully used molecular detection of *Nosema* in pools of 5 bees/colony for large-scale colony screening of *Nosema* infections [40], [42], larger pools (25–30 bees) or repeated measurements may have increased the sensitivity of this screen at the individual colony level, though likely not at the apiary level.

**Table 1:** Primers used for molecular analysis for identification of bee populations, pathogens and parasites.

Primer	Forward Sequence (5′-3′)	Reverse Sequence (5′-3′)	Product Size (bp)	Reference
**ABPV**	TTATGTGTCCAGAGACTGTATCCA	GCTCCTATTGCTCGGTTTTTCGGT	900	Benjeddou et al. 2001
**BQCV**	TGGTCAGCTCCCACTACCTTAAAC	GCAACAAGAAGAAACGTAAACCAC	700	Benjeddou et al. 2001
**CBPV**	AGTTGTCATGGTTAACAGGATACGAG	TCTAATCTTAGCACGAAAGCCGAG	455	Ribiere et al. 2002
**DWV**	ATCAGCGCTTAGTGGAGGAA	TCGACAATTTTCGGACATCA	701	Chen et. al. 2005
**IAPV**	GCGGAGAATATAAGGCTCAG	CTTGCAAGATAAGAAAGGGGG	586	Di Prisco et. al. 2011
**KBV**	GATGAACGTCGACCTATTGA	TGTGGGTTGGCTATGAGTCA	417	Stoltz et al 1995
**SBV**	GCTGAGGTAGGATCTTTGCGT	TCATCATCTTCACCATCCGA	824	Chen et. al. 2005
**Nosema SSU**	GCCTGACGTAGACGCTATTC	GTATTACCGCGGCTGCTGG	400	Klee, Besana et. al. 2007
**Mitochon. Markers**	TGATAAAAGAAATATTTTGA	GAATCTAATTAATAAAAAA	688	Arias and Sheppard 1996

Abbreviations: Israeli acute paralysis virus (IAPV), acute bee paralysis virus (ABPV), black queen cell virus, (BQCV), chronic bee paralysis virus (CBPV), deformed wing virus (DWV), kashmir bee virus (KBV), and sacbrood virus (SBV). References: Arias MC and WS Sheppard WS (1996) *Molecular Phylogenetics and Evolution* 5: 557–566; Benjeddou et al. (2001) *Applied and Environmental Microbiol*ogy 67:2384–2387; Chen et al. (2005) *Applied and Environmental Microbiol*ogy 71(1):436–441; Di Prisco et. al. (2011) *Journal of General Virology* 92: 151–15; Klee et al. (2007). Journal of Invertabrate Pathology 96: 1–10. Ribiere et al. (2002) *Apidologie* 33: 339–351; Stoltz et al. (1995) *Journal of Apicultural Research* 34: 153–160.

### Survey of Viruses

Pools of 5 foragers/colony were collected into RNAlater (Qiagen, Valencia, CA); all 81 colonies in the survey were assayed. RNA was extracted from these pooled samples using Tri-Reagent (Sigma-Aldrich, St. Louis, MO). cDNA was synthesized using 150 ng of RNA for each pooled samples. The presence of seven common honey bee viruses was identified using PCR with previously published primers specific for each virus [37], [43]–[46]. See Table 1 for a listing of the viruses, primers used, and references for the primers. PCR conditions were as follows: 2 minutes at 95°C, followed by forty cycles of 95°C (30 s), 55°C (60 s), and 68°C (120 s) and a final extension at 68°C for 7 minutes. PCR products were separated on a 1% agarose gel and visualized with ethidium bromide. Bees collected from apiaries at Penn State were used as positive controls. A previous large-scale survey of viral infection dynamics in colonies found that molecular analysis of pools of five foragers/colony provided equivalent detection sensitivities as individual analyses of 10–15 bees per colony [14]; however, as in the case of the survey for *Nosema* above, larger pools (25–30 bees) or repeated measurements may have increased the sensitivity of this screen at the individual colony level, though likely not at the apiary level.

Associations between viral diversity (the number of viruses present in a colony) and colony size (measured by the number of frames of adult bees) were assessed in all colonies where colony size measurements were available. Fifteen apiaries (sites 1, 2, 3, 4, 5, 6, 9, 10, 11, 12, 13, 14, 15, 20, and 21) with 58 colonies included in the analysis. A Kruskal-Wallis test was used to determine if there were significant differences in colony size among colonies with 0, 1 or 2 viruses using JMP 9.0.2 (SAS, Cary, NC).

Associations between viral diversity and *Varroa* levels were measured in the 19 apiaries (see above) in which *Varroa* measurements were available; 66 colonies were used. A Kruskal-Wallis test was used to determine if there were significant differences in *Varroa* loads among colonies with 0, 1, or 2 viruses, followed by a pairwise comparisons using nonparametric Wilcoxon pairwise tests, using JMP 9.0.2 (SAS, Cary, NC).

Because it was not possible to determine which variable (viral diversity versus number of frames or number of *Varroa*) is dependent and which is independent, we also performed a correlation analysis in JMP 9.0.2 (SAS, Cary, NC). To obtain a normal distribution of the data, viral diversity were converted to logarithmic scale using the following equation: log number of viruses  = log_10_ (number of viruses +1).

### Survey of Hygienic Behavior

When possible, a colony's hygienic behavior (the removal of freeze-killed pupae) was assessed as in [47]. A 3-inch (7.62 cm) diameter PVC cylinder was pressed into a frame of capped brood containing purple-eyed pupae; this area corresponds to approximately 207 cells of naturally drawn African honey bee comb. The percent hygienic behavior for a colony was calculated by taking the final number of fully and partially removed pupae/(207 – number originally uncapped or empty cells) *100 (Table S1).

Hygienic behavior was assessed for 10 apiaries, at sites 1, 3, 4, 11, 12, 13, 14, 15, 20, and 21. In total, 36 colonies were assayed. Associations between hygienic behavior and elevation, colony size, and log *Varroa* counts were determined using a correlation analysis with JMP 9.0.2 (SAS, Cary, NC). A Kruskal-Wallis test was used to determine if there were differences in levels of hygienic behavior among colonies with 0, 1 or 2 viruses, followed by nonparametric Wilcoxon pairwise tests. A correlation analysis was also performed between the log number of viruses and hygienic behavior.

### Subspecies Identification

Heads of foragers were dissected and homogenized with a Fastprep instrument (Thermo Fisher, Waltham, MA) for three cycles at maximum time and speed. DNA was extracted using the DNeasy Blood and Tissue Kit (Qiagen, Valencia, CA) according to manufacturer's instructions. Mitochondrial DNA including the tRNA ILE and part of the ND2 gene were amplified as described in [48], see Table 1 for primer sequences. PCR was performed on a Mastercycler Pro (Eppendorf, Hauppauge, NY) using 25 µl reactions consisting of 2.5 units of platinum Taq DNA Polymerase, PCR buffer minus magnesium at a concentration of 1X, 0.2 mM dNTP mix, 1.25 mM MgCl_2_, 5% DMSO, 0.2 µM primers and 10 ng of extracted DNA; reagents were purchased from Invitrogen (Carlsbad, CA). The PCR was carried out using the thermal profile of 1 minute at 94°C, followed by 40 cycles of 94°C (40 s), 42°C (80 s) and 62°C (120 s) and a final extension at 72°C for 4 minutes. No-template controls were performed with each PCR run. Products were visualized on 1.0% agarose gels, excised and extracted with the QIAquick PCR Purification Kit (Qiagen, Valencia, CA) and submitted for sequencing. Sequencing was preformed at the Genomics Core Facility at Pennsylvania State University. Sequences were aligned using ClustalW [49] package in BioEdit 7.6 [47]. There were 24 unique haplotypes from the 109 individuals sequenced (see Table S3 for the haplotype designation for each individual). These 24 unique haplotypes were used to construct a neighbor-joining tree [50] along with haplotypes corresponding to the subspecies described from [48](see Figure S1 for the tree). The ND2 region variable sites are described in Table S4.

### Survey of Pesticides

When possible, brood nest wax and bee bread (stored, fermented pollen mixed with nectar) were collected for pesticide analysis. Approximately 3–10 grams of each matrix was collected into individual sterile, 50 ml centrifuge tubes for each colony. Samples were shipped to Penn State University within three weeks of collection and stored at −80°C. Pesticide analysis was performed on wax (45 colonies) and pollen (25 colonies) samples obtained from 15 (sites 1–15) and 13 (sites 1, 2, 4, 5, 6, 8, 9, 10, 11, 12, 13, 14, 15) of the apiaries respectively. One to five wax or pollen samples were pooled by site to provide a single wax and single pollen sample per site for analysis, averaging 9.45 and 7.1 grams/pooled sample respectively. Samples were shipped to the USDA-AMS-NSL lab in Gastonia, NC for extraction and were screened for the presence of 171 pesticides and toxic metabolites by LC-MS-MS and GC-MS according to methods described in [51].

## Results

### Varroa

**Figure 2:**
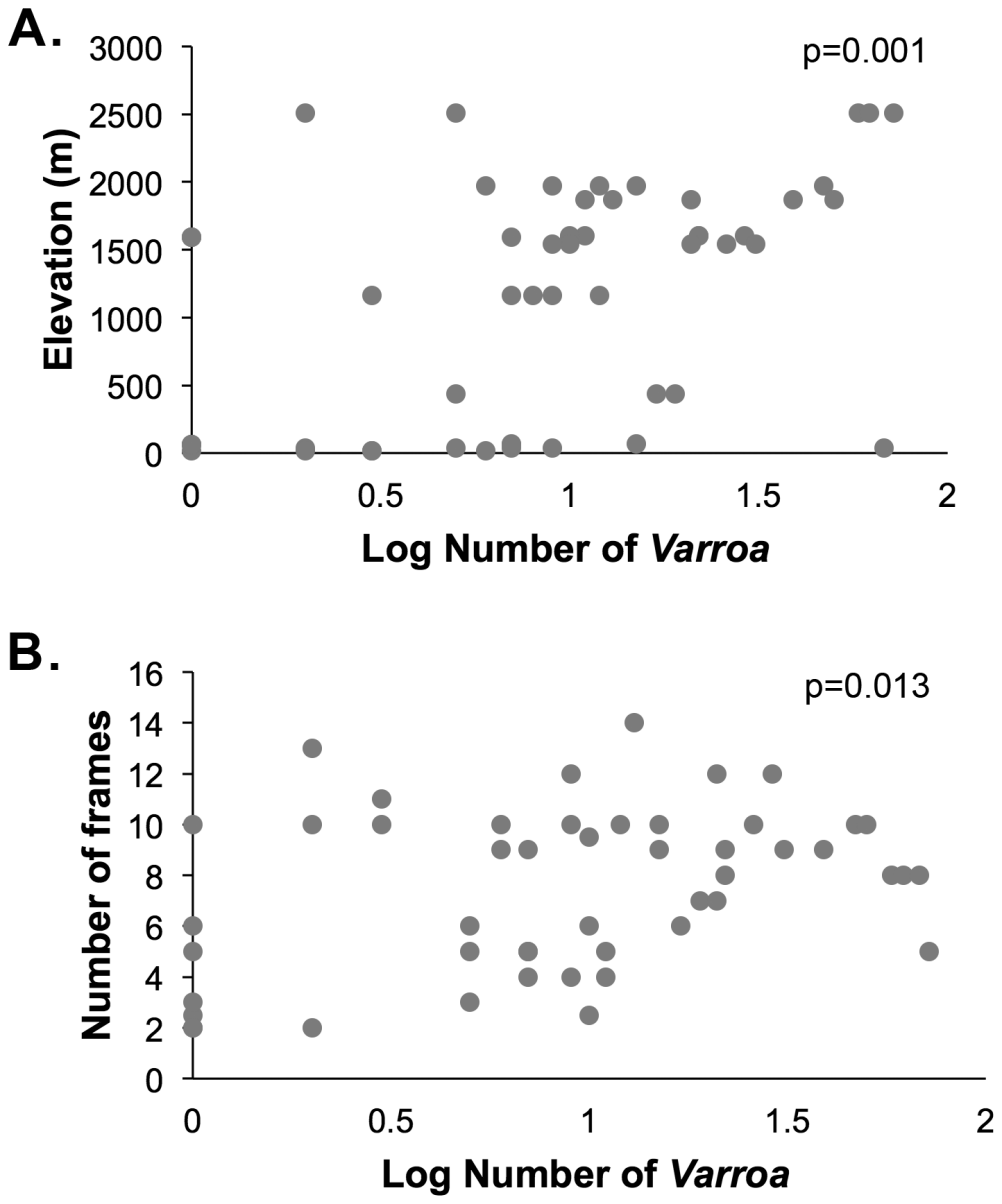
Association of *Varroa* infestation with elevation and colony size. **A**. Levels of *Varroa* mites were positively correlated with elevation, with colonies at higher elevations having significantly higher average numbers of *Varroa* (r(53) = 0.44, p = 0.001). **B**. Levels were also positively correlated with colony size ((48) = 0.35, p = 0.013). *Varroa* counts were converted to logarithmic scale.

Of the 19 apiaries assayed for *Varroa* mites, 17 (89.5%) had mites present (Table S1). The two assayed apiaries (sites 17 and 18) that did not have *Varroa* were in far northeastern corner of Kenya near the border with Somalia and Ethiopia. In total, 66 colonies were assayed for *Varroa*, and *Varroa* was found in 55 (83%) of them. The levels of *Varroa* were highly variable across colonies and apiaries. Twenty-four (corresponding to 36%) colonies had 5 or fewer mites in samples of ∼350 bees, 16 colonies had 6–10 mites, 11 colonies had 11–20 mites, 6 colonies had 21–30 mites, while 9 colonies had more than 30 mites. In the US, it is recommended that colonies are treated for mites when 5–20 mites are found in samples of ∼300 bees in the fall [39], [52]. *Varroa* levels were positively correlated with elevation (r(53) = 0.44, p = 0.001; Figure 2A) and with colony size (r(48) = 0.35, p = 0.013, Figure 2B). There was no correlation between colony size and elevation (r(48) = −0.02, p = 0.87, data not shown)

Since *Varroa* levels can change seasonally, we repeated this analysis using only colonies sampled in June 2010 (from apiaries 1, 3, 4, 10, 11, 12, 13, 14, 15,). Again, colonies at higher elevations had significantly higher *Varroa* loads (r(38) = 0.39, p = 0.014; data not shown), and there was a trend for a positive correlation between *Varroa* levels and colony size (r(38) = 0.31, p = 0.054, data not shown).

### Nosema

Sixteen apiaries were assessed for the presence of *Nosema ceranae* and *Nosema apis*. *Nosema* was identified in 4/5 colonies at site 12, in 5/5 colonies at site 13, in 4/5 colonies at site 15, and in 2/3 colonies at site 22 (Table S1). An RFLP approach was used to determine the presence and subspecies of *Nosema* [40]. Interestingly, the fragmentation pattern was not consistent with either species. The 400 bp 16S rRNA gene amplicon from these samples showed an alternate cleavage pattern when double digested with restriction enzymes *Msp*I and *Nde*I. A digestion pattern of 3 fragments (one at 225 bp, one at 100 bp and one at 75 bp) was observed. The predicted digestion pattern for *Nosema apis* of three fragments (at 175, 136, and 91 bp) was observed for the *N. apis* control. The predicted digestion pattern for *Nosema ceranae* of 2 fragments (175 and 225 bp) was observed for the *N. ceranae* control. Sequencing of the 400 bp *Nosema* 16S rRNA gene region used for the RFLP analysis revealed two recombination events. An inversion of TAC from CAT at position 151 removed the predicted *NdeI* cleavage site from the reference and the *N. apis* control sequences. An insertion of a thymine into the sequence CATAG produced an alternate *NdeI* cleavage site of CATATG at position 341. The *MspI* enzyme cleaved the sequence CCGG as expected at position 242. The amplicons were not cleaved by the enzyme *PacI* which exploits a unique digestion site for *Nosema ceranae*.

### Viruses

All colonies in all 24 apiaries were assessed for the presence of seven viruses commonly found in honey bees in North America and Europe: Israeli acute paralysis virus (IAPV), acute bee paralysis virus (ABPV), black queen cell virus, (BQCV), chronic bee paralysis virus (CBPV), Deformed wing virus (DWV), Kashmir bee virus (KBV), and sacbrood virus (SBV). Only DWV, BQCV, and ABPV were detected in Kenyan bee populations. Viruses were found in 20 apiaries; no viruses were detected in apiaries at sites 15, 16, 17, and 18 (Table S1). Sites 16, 17, and 18 are in northeastern Kenya (which were also free of *Varroa*), while site 15 is near the southeastern coast. DWV was found at 12 sites in 36% (29 out of 81) of the colonies, BQCV was found at 18 sites in 60% (49 out of 81) of the colonies, and ABPV was found in one colony at Site 13 (this colony also had DWV and *Nosema* infections).

**Figure 3:**
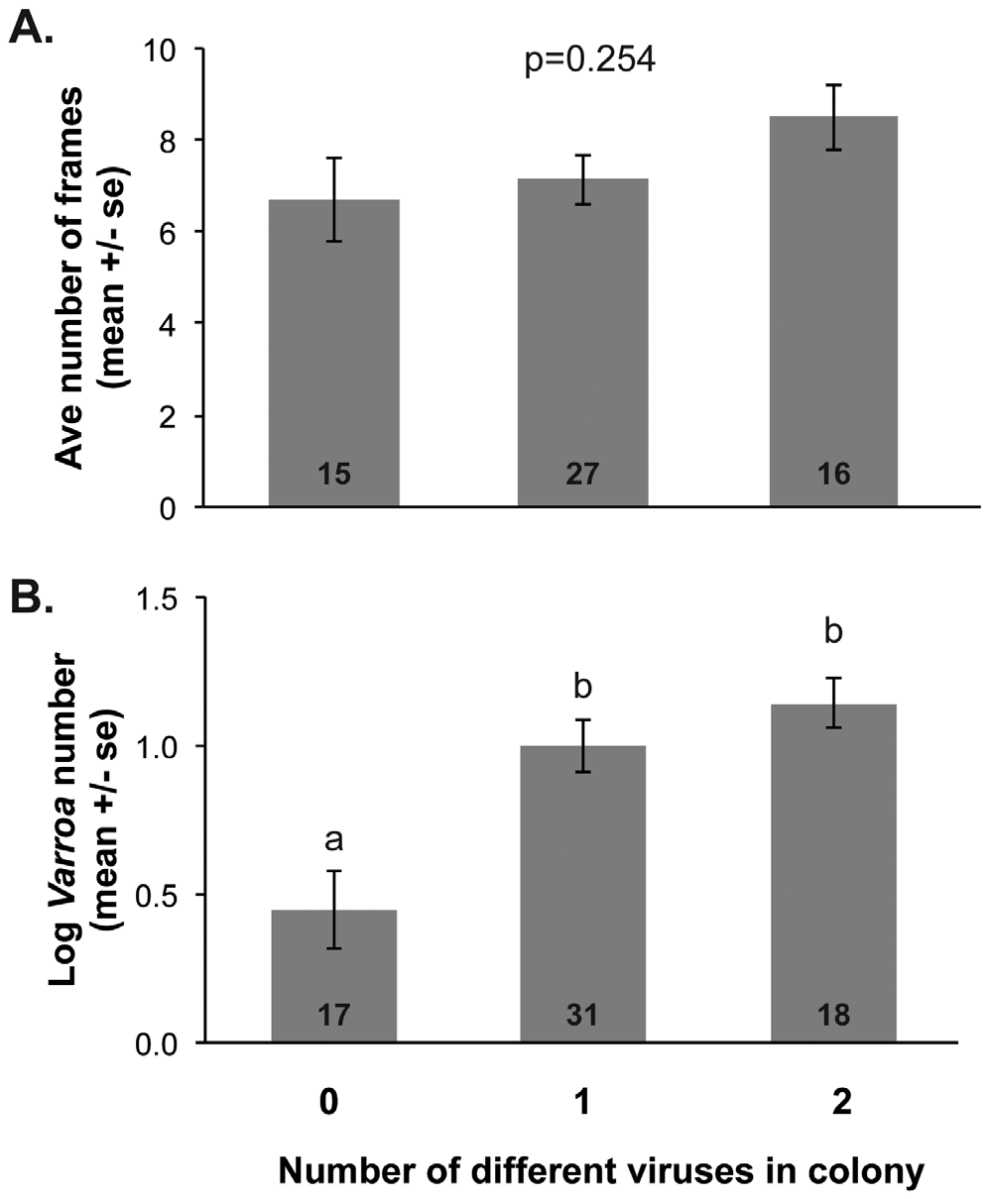
Association of viral diversity with colony size and *Varroa*. **A**. Colony size (the number of frames of bees) was not affected by viral diversity (the number of viruses in a colony), H(2) = 2.74, p = 0.254. **B**. However, colonies with different number of viruses had significantly different numbers of *Varroa* (H(2) = 13.10; p = 0.0014). Colonies with 1 or 2 viruses had significantly higher *Varroa* loads than colonies that had no viruses (p<0.05, Wilcoxon pairwise tests, different letters denote significant differences). The number of colonies in each group is indicated at the bottom of each bar. *Varroa* counts were converted to logarithmic scale.

Average colony size (based on the number of frames of bees) was not associated with viral diversity (the number of viruses in a colony; Kruskal-Wallis: H(2) = 2.74, p = 0.254, Figure 3A). A correlational analysis between colony size and viral diversity was also not significant (r(57) = 0.20, p = 0.128). However, there was a significant correlation between viral diversity and *Varroa* loads (Kruskal-Wallis: H(2) = 13.10; p = 0.001); colonies with 1 or 2 viruses had significantly higher *Varroa* loads than colonies that had no viruses (p<0.05, Wilcoxon pairwise tests, Figure 3B). Similarly, a correlational analysis between *Varroa* loads and viral diversity was significant (r(65) = 0.49, p<0.001). Eight out of 66 colonies with *Varroa* infestations had no viruses, while only 2 colonies had one virus and no detectable *Varroa*.

Since levels of viruses can change over time [14], we repeated this analysis using colonies sampled only in June 2010 and obtained similar results. There was no correlation between viral diversity and colony size (r(47) = 0.11, p = 0.456, data not shown), but there was a significant positive correlation between *Varroa* levels and viral diversity (r(47) = 0.33, p = 0.023, data not shown).

### Hygienic behavior

**Figure 4:**
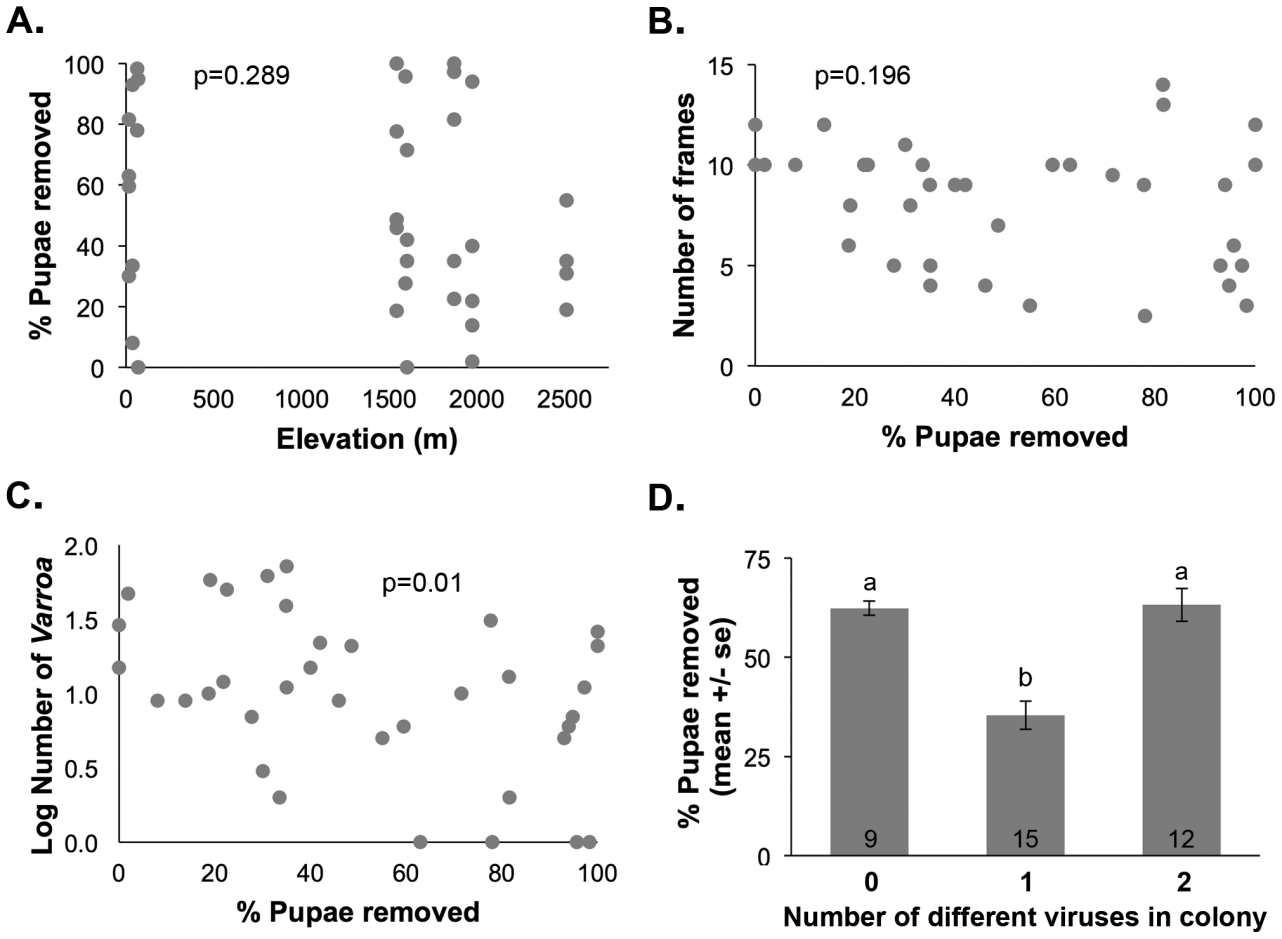
Association of hygienic behavior with colony location, size, parasite and pathogen loads. **A**. There were no significant correlations between hygienic behavior and elevation (r(35) = −0.18, p = 0.289). **B**. Hygienic behavior was not correlated with colony size (r(35) = −0.22, p = 0.196). **C**. There was, however, a significant negative correlation between hygienic behavior and the numbers of *Varroa* in these colonies (r(35) = −0.42, p = 0.011; *Varroa* counts were converted to logarithmic scale). D. Hygienic behavior was also significantly associated with viral diversity (H(2, 34) = 6.43, p = 0.040); levels were lower in colonies with 1 type of virus versus colonies with 0 or 2 viruses (p<0.05, Wilcoxon pairwise tests, different letter denote significant differences). The number of colonies in each group is indicated at the bottom of each bar.

Hygienic behavior was assessed in 36 colonies from 10 sites (Table S1). There were no significant correlations between hygienic behavior and elevation or colony size (r(35) = −0.18, p = 0.289, Figure 4A and r(35) = −0.22, p = 0.196, Figure 4B, respectively). There was, however, a significant negative correlation between hygienic behavior and the numbers of *Varroa* in these colonies (r(35) = −0.42, p = 0.011, Figure 4C). Hygienic behavior was also significantly associated with viral diversity (Kruskal-Wallis: H(2, 34) = 6.43, p = 0.040); levels were lower in colonies with 1 type of virus versus colonies with 0 or 2 viruses (p<0.05, Wilcoxon pairwise tests, Figure 4D). Note the correlational analysis of viral diversity and hygienic behavior was not significant in this case (r(35) = −0.0277, p = 0.90). Similar results were obtained if only colonies sampled in June 2010 were used (elevation: r(25) = −0.31, p = 0.122; number of *Varroa*: r(25) = −0.63, p = 0.0005; viral diversity: H(2) = 5.55, p = 0.076, r(25) = −0.21, p = 0.292; data not shown), though in this case the negative correlation between hygienic behavior and colony size was significant (r(25) = −0.46, p = 0.019).

### Pesticides

Pesticide analysis was performed on pool wax samples from 15 sites (1 sample per site) and pooled bee bread samples from 13 sites. Samples were screened for the presence of 171 pesticides and toxic metabolites. Only 4 pesticides, 1-naphthol, chlorothalonil, chlorpyrifos and fluvalinate, were identified and were present at very low levels (below 50 ppb) with the exception of 1-napthol which was found at only one location (site 9, Nji-ini Forest) at 116 ppb (Table S1). However one or more of these pesticides were detected at 14 of the 15 sites sampled (data not shown).

### Subspecies

Our attempt to identify subspecies of *A. mellifera* in Kenya was based on the work of Arias and Sheppard (1996) (see Table S1 for haplotype information and Figure S1 for the neighbor-joining tree. Accession Numbers KJ628964-KJ628987). Representative haplotype 3.4.11 is identical in sequence to the previously described *A. m. monticola* (Figure S1). Our analysis suggests *A. m. monticola* is present at high altitude on Mount Elgon as previously suggested by Ruttner [27], [53] with some mixing lower on the mountain at Chepkui, but ND2 *monticola* is also present in the Aberdares at Marchorwe. However it is important to note that other bees from all three sites exhibit *A. m. scutellata* haplotypes. The *monticola* clade forms a sister clade to a *scutellata* group (Group A) that includes 15 apiaries from across the Kenya, excluding the northern region and the southeast. This ND2 region is identical to that from honey bees from South Africa (SCUTE2) and *A. m. adansonii* (ADANS1) from Nigeria, and similar to Africanized bees from Brazil (BRASI1 and BRASI2). A distinct clade of *scutellata* honey bees (Group B) with the dominant haplotype of 2.4.11 (identical to SCUTE1 from Kenya) is found predominantly in the central and eastern portion of the country. The individuals in the haplotype group identified by 12.1.12 are from the coast and the far north of Kenya (identical ND2 regions are found at sites 12, 13, 16, 17). Apiary 12 is in the Arabuko Sokoke Forest Reserve, a remnant of the coastal tropical forest that supports a number of unique endemic species. Although no molecular comparison is available from other studies, we speculate that this could be *A. m. litorea* given the close relationship with *A. m. lamarckii* (LAMARC) and the honey bees in the northern apiaries at Mandera [27]. Individuals from one colony at apiary 18, called Mandera West, were distinct from the other Kenyan bees, although another colony at the same apiary groups with *scutellata* A. These could represent either *A. m. yemenitica* or the recently described *A. m. simensis* [27], [28], neither of which have been described with molecular makers. There were 14 unique individual haplotypes whereas many apiaries had multiple ND2 haplotypes (Table S3). We found no correlation between genotype, as measured the ND2 region, and honey bee health although more data will be require to clearly distinguish populations or subspecies.

## Discussion

Our survey suggests that several new parasites and pathogens (*Varroa*, *Nosema*, DWV, BQCV, and ABPV) have recently invaded honey bee populations in East Africa (see below for further discussion). However, none of these factors are correlated with colony size, implying that several factors thought to critically undermine bee populations in the US and Europe (*Varroa*, *Nosema*, and pesticide use) are not yet directly impacting Kenyan bee populations in terms of this metric. However, since there may be a time-lag before newly introduced parasites and pathogens cause substantial negative effects, continuous monitoring of these populations should be conducted to evaluate the long-term dynamics of these host-pathogen interactions. Our data also suggests that chemical control methods for *Varroa* and *Nosema*, which are heavily used by US beekeepers, may be unnecessary at this time for Kenyan honey bees, and indeed, these bees may possess novel resistance mechanisms. Furthermore, we found an intriguing correlation between elevation and *Varroa* levels, suggesting that environmental factors (climate, landscape ecology) may play a key role in mediating this host-parasite interaction, and perhaps honey bee health in general, though the effect of these environmental factors needs to be explored in greater detail in larger scale studies. We did not find any association with subspecies genotype and honey bee health. However, based on the molecular marker that we used (the ND2 region of the mitochondria) there was considerable gene flow between populations, and thus more sensitive markers may be needed to fully characterize subspecies and population differences.

*Varroa* alone does not appear to strongly impact honey bee colonies in Kenya. Based on recommendations for US beekeepers, approximately 2/3 of the surveyed colonies had *Varroa* levels high enough to warrant treatment to control mite populations [39], [52]. However, *Varroa* levels in Kenyan colonies are not correlated with decreases in colony strength; in fact, there is a positive correlation between *Varroa* numbers and colony size. Colonies with higher levels of hygienic behavior did have lower levels of *Varroa*, as expected [54], but hygienic behavior was quite variable across the surveyed colonies. *Varroa* levels were strongly positively correlated with elevation. Based on a limited preliminary study of the impacts of geographic location on colony weight and *Varroa* levels (see Figure S2) this is possibly due to differences in climate or floral resources rather than due to differences in honey bee genetic background, though the association between nutrition, climate and pathogen loads in honey bee populations needs to be more thoroughly assessed. *Varroa* was recently found in honey bee colonies in Nigeria as well, at comparable levels (approximately 80% of colonies infested, with 2–55 mites found on 100 bees), but again no negative impacts on *Varroa* infestation on colony health and productivity were reported [55]. It is unclear what factors contribute to reduce the impacts of *Varroa* on African bees relative to European bees. Previous studies have indicated that Africanized bees in South America have higher levels of hygienic behavior, higher levels of grooming mites off of adult bees, lower levels of mite reproduction on pupae, and are less attractive to *Varroa* mites than European bees [56]. African bee subspecies also tend to abscond (abandoning hives and sometimes migrating) and swarm (where a large fraction of the colony leaves with the queen to form a new colony) more readily than European bees [27], thereby causing breaks in brood rearing that may reduce *Varroa* loads [57]. Indeed, breaking the brood cycle is recommended as a method to reduce *Varroa* loads in European honey bees [58], and removal of drone brood significantly reduces *Varroa* levels [59]. However, all of these factors presumably would lead to lower overall *Varroa* levels in colonies, rather than a reduced impact on worker mortality and colony size with equivalent levels. Thus, other parameters, perhaps physiological or behavioral, may contribute to the higher levels of tolerance of African bees to *Varroa* infestation.

Kenyan bee populations also displayed infections with DWV, BQCV, and ABPV, and *Varroa* numbers are strongly positively correlated with viral diversity (number of viruses present). Previous studies have demonstrated that *Varroa* can vector DWV and IAPV [37], [38], and the introduction of *Varroa* to a naïve population of honey bees in Hawaii was correlated with reduction in sequence diversity of DWV [60], suggesting *Varroa* mites vector a specific viral strain. Thus, *Varroa* may have introduced these three viruses to the Kenyan honey bee populations, or is altering the population structure of these viruses. Alternatively, the presence of these viruses may be weakening bees' defenses to *Varroa*; this possibility has not yet been examined. Interestingly, a 2010 study of honey bee colonies in Uganda (which borders western Kenya and was thought to be *Varroa*-free at the time of the study) found BQCV in 30–40% of the colonies, but DWV and ABPV were not detected [61], suggesting that these two viruses may have been recently introduced to Kenya, perhaps by *Varroa*. Indeed, the viruses were not present in the geographically distant apiaries in far northeastern Kenya, suggesting that they, and *Varroa*, have not yet spread to this region. It should be noted that while the number of individual bees assayed for viruses in each colonies was small (5 bees), the number of colonies assayed throughout Kenya (81 colonies) and those assessed for correlation between viral diversity and *Varroa* levels (66 colonies) was fairly substantial. Thus, while the results of the individual colonies should be interpreted with caution (see Pirk et al [62] for more information on pathogen sampling in bee populations), the overall data set suggests that only three common European viruses are circulating, at this point, in the Kenyan populations at detectable levels, and that these are associated with *Varroa* parasitization levels.

As is the case with *Varroa*, DWV and BQCV do not appear to negatively impact Kenyan bee colonies, since viral infection is not correlated with colony size. Kenyan colonies are overall relatively small (7 frames of honey bees on average, compared to US colonies which can easily reach 20+ frames) and thus may not be at their limits of productivity. Better measures of the impacts of viruses, *Varroa*, and *Nosema* on bee health would include the longevity of infected and uninfected colonies, their ability to reproduce and successfully establish new colonies. Indeed, other studies have indicated that colonies may be particularly sensitive to the impacts of viruses during specific stressful periods (such as colony founding or migration) which could result in reduced populations. In European honey bees, high DWV levels in the fall are associated with reduced overwintering survival [63], [64]. While there was an effect of viral diversity on hygienic behavior, this was not consistent – colonies with no viruses were as hygienic as colonies with two viruses. Previous studies have suggested that bees more readily remove larvae that are parasitized by *Varroa* with high viral titers [65], but our results suggest that hygienic behavior is not likely functioning as a major mechanism to reduce viral loads in Kenyan colonies.

The gut microsporidia *Nosema ceranae* was linked to colony losses in Spain [66], and high levels of *Nosema bombi* have been associated with declining bumble bee populations in North America [67]. However, other studies have not found a strong correlation with *Nosema* presence and colony declines [14], [24], suggesting that *Nosema* may act in concert with other factors, such as pesticides [68], to reduce bee health. While *Nosema* was previously found in Zimbabwe and South Africa [69], [70], it was not detected in a previous survey in 1996–1998 in Kenya ([36]; Shi Wei, personal communication, 2011). In our 2010 survey, *Nosema* was only identified in two apiaries at the coast [site 12 and 13], at site 15 near the Tanzania border and, surprisingly, at high elevation interior site on Mt. Elgon (site 22). Using PCR-based detection methods we did not detect *Nosema* in other locations. As in the case of the viral analyses, though the sample sizes for individual colonies was limited (5 bees/colony), our analysis at the apiary level should be quite robust in detecting *Nosema* if it is present. Notably, colonies in coastal *Nosema*-infected apiaries were similar in size to those in non-infected apiaries, and *Varroa* levels were substantially lower than other regions in the country. Thus, *Nosema* does not appear to be affecting honey bee populations in Kenya.

In the US, >90% of honey bee colonies contain pesticide residues [51]. Over 129 different pesticide-related chemicals have been found in US bee colonies, with an average of 6 chemicals per colony. Pesticide exposure has been linked to honey bee population declines [10], [71] reduced survival and impaired development of brood [72], impaired cognitive function, [73]–[75], altered expression of immune genes [76], and increased *Nosema* loads [68], [77]. In the Kenyan colonies surveyed in this study we found only four pesticides, and most were at very low levels compared to pesticide levels in North America. The most commonly found pesticide was the fungicide chlorothalonil (12 of 15 sites), while chlorpyrifos, an organophosphate insecticide, was found in five of the apiaries tested. Interestingly, fluvalinate was found in one apiary: fluvalinate is commonly used to control *Varroa* mites in the North America and Europe; however this broad-spectrum pyrethroid is also used to control mosquitos and horticultural pests such as aphids, whiteflies and thrips. The low levels of pesticides in hives from across Kenya, particularly when compared to levels in developed countries, suggests pesticide residues play only a limited role in honey bee health in Kenya at this time.

## Conclusions

Honey bees provide critical pollination services to agriculture and natural landscapes, and the honey and wax produced by honey bees represent a potential source of income for families in East Africa and across the world. Our survey suggests that several new parasites and pathogens (*Varroa*, *Nosema*, DWV, BQCV, and ABPV) have recently invaded honey bee populations in East Africa. Our results indicate that these parasites and pathogens are not yet impacting honey bee health in Kenya, at least in terms of colony size. Interestingly, levels of *Varroa* are strongly impacted by elevation/geographic region, suggesting that environmental factors modulate *Varroa* infestation rates. Finally, our phylogenetic analyses suggest there is considerable mixing of honey bee populations in Kenya, and thus newly introduced parasites and pathogens can likely move easily throughout the region. Our studies suggest that honey bee populations in East Africa appear to be largely resistant or tolerant of the parasites and pathogens that threatened honey bee populations in other parts of the world, and are not yet significantly impacted by other stressors, such as exposure to environmental toxins. However, since there may be a time lag before these newly introduced pathogens and parasites significantly impact honey bee populations, additional long-term monitoring is necessary. Finally, our results also highlight the importance of environmental factors in buffering honey bee populations from these stressors, and with increasing habitat fragmentation and destruction and environmental extremes brought on by global climate change, populations of this keystone species, in Africa and throughout the world, will be under increasing pressures.

## Supporting Information

Figure S1 **Neighbor-joining tree (Saitou and Nei, 1987) comparing representative Kenyan honeybee haplotypes (see Table S2) with subspecies described in Arias and Sheppard 1996 (in capitals).** The European subspecies *Apis mellifera mellifera* (MELLI1) is used as the outgroup. The percentage of replicate trees in which the associated taxa clustered together in the bootstrap test (2000 replicates) are shown next to the branches for values greater than 30% (1). Branch length indicates number of SNP differences. The analysis involved 39 nucleotide sequences from the ND2 mitochondrial region. Twenty-four of the 39 are representative unique haplotypes. For a list of all individuals represented by these haplotypes see Table S2. There were a total of 579 nucleotide positions in the final dataset. Phylogenetic analysis was conducted in MEGA5 (2). This analysis suggests as many as 7 clades of *A. mellifera* within Kenya. Whether this is evidence of more subspecies than previously described will require more sampling. Moreover, the weak statistical support suggests a need to expand beyond the ND2 region to describe East African subspecies of *A. mellifera*. Accession Numbers KJ628964-KJ628987.
(DOCX)

Figure S2 **Effect of location on colony weight and** ***Varroa*** **numbers.** Colonies of from upland site 1 (icipe) were assayed in June 2010, immediately prior and after moving upland colonies from site 1 to site 25 on the coast. Coastal colonies were assayed at nearby apiaries (sites 26 and 27). There were no significant differences in colony weight (**A**, F(3,26 = 1.43), p = 0.256), but there were significantly fewer *Varroa* in colonies moved to site 25 (**B**, F(3,25) = 6.63, p = 0.0019). In August 2010, upland colonies at sites 1 and 25 and coastal colonies at site 25 were assayed. There were again no significant differences in weight (**C**, F(2, 16) = 1.29, p = 0.301), but both upland and coastal colonies had significantly fewer *Varroa* at site 25 (**D**, F(2,16) = 6.96, p = 0.0067). The number of colonies in each group at each timepoint is indicated at the bottom of each bar in B and D. Letters represent groups that were significantly different with a Tukey HSD post-hoc pairwise comparison, at p<0.05. While the graphs show the actual average numbers of *Varroa*, counts were converted to logarithmic scale for statistical analysis.
(DOCX)

Table S1 **Apiary colony list indicating location,** ***Varroa*** **load, hygienic behavior, presence of virus,** ***Nosema*** **, pesticide and subspecies identification.** Each apiary was given a site number and apiary name. Each colony has a unique identification number with the apiary number in the first position and the colony number in the second. Colony size is indicated by numbers of frames with bees. Total *Varroa* counts are based on standard sugar roll assay described in Ellis and Macedo, 2001 (1). The percent hygienic behavior (2) was calculated by taking the final number of fully and partially removed pupae/(207 – number originally uncapped or empty cells) *100. Boxes with UD are undetermined indicating measurements were not taken for these colonies. Positive for virus or *Nosema apis* detection per colony is indicated by an “X”. The presence of pesticide detected in wax or bee bread is indicated in parts per billion (ppb). Only four pesticides were detected: CP (chloropyrios), CT (chlorothalonil), N (1-naphtol), F (fluvalinate). ND2 subspecies identification is based on the analysis presented in Figure S1. Individuals from most of the colonies grouped with the scutellata A or B clades. Some were identical in sequence to *A. mellifera monticola* described by Arias and Sheppard, 1996 (3). Some individuals from colonies at sites 12, 13, 16 and 17 were most closely related to *A. mellifera lamarckii* and are thus called “lamarckii-like”. Colonies at site 18 were unique and perhaps represent a distinct subspecies (*A. m. simensis* or *yemenitica*). A few individuals did not group with any previously described subspecies and are thus indicated as UD. Interestingly three colonies (2.1, 2.2, and 16.1) had individuals from multiple mitochondrial lineages.
(DOCX)

Table S2 **Type of data collected at each apiary.** An “X” indicates the data type at the top of the column was collected in at least one colony in the corresponding apiary. See Materials and Methods for a full description of the type of data collected and the analyses that were performed.
(DOCX)

Table S3 **A list of ND2 haplotypes used for comparison.** Individuals were sequenced for the ND2 region (as described in 1) from 24 apiaries across Kenya. There were 24 unique haplotypes from 109 individuals sequenced. Each column is headed by the representative haplotype (in bold) used for analysis. Haplotypes identical to the representative type follow in each column. The columns are the far right is a list of unique single haplotypes. Numeric designations for each individual follow the following scheme: apiary.colony.individual.
(DOCX)

Table S4 **ND2 region variable sites from Kenya honeybee haplotypes compared to the subspecies** ***A. m. adansonii*** **(ADANS2) (1).** The nucleotide positions starting from the ND2 ATC (isoleucine) are indicated in the top row with corresponding position numbers from the complete honeybee mitochondrial genome (2). The codon position for each SNP is indicated at the bottom of the figure. Over the 579 bp of the ND2 coding region 85% of SNPs were in the third codon position. Three SNPs were in the second codon position. The first is a transversion at position 53 (T↔A) that results in an amino acid change of isoleucine ↔ asparagine. A second codon position change is a transition at nucleotide position 161 (C↔T) resulting in an amino acid change of threonine ↔ isoleucine. The third second codon change (position 458) is also a transition (C↔T) resulting in threonine ↔ isoleucine. The Kenya honeybee population also shows a first codon transition (position 412; G↔A) that results in an amino acid difference (valine ↔ isoleucine) when compared to the reference sequence.
(DOCX)

Form S1 **Collaborating authories.**
(DOCX)
