# Supplementary material for: Evaluation of the Distribution and Impacts of Parasites, Pathogens, and Pesticides on Honey Bee (Apis mellifera) Populations in East Africa
Source: PLoS One. 2014 Apr 16;9(4):e94459. doi: 10.1371/journal.pone.0094459 (PMC3989218; doi:10.1371/journal.pone.0094459)
Supplement: Figure S1 — Neighbor-joining tree (Saitou and Nei, 1987) comparing representative Kenyan honeybee haplotypes (see Table S2) with subspecies described in Arias and Sheppard 1996 (in capitals). The European subspecies Apis mellifera mellifera (MELLI1) is used as the outgroup. The percentage of replicate trees in which the associated taxa clustered together in the bootstrap test (2000 replicates) are shown next to the branches for values greater than 30% (1). Branch length indicates number of SNP differences. The analysis involved 39 nucleotide sequences from the ND2 mitochondrial region. Twenty-four of the 39 are representative unique haplotypes. For a list of all individuals represented by these haplotypes see Table S2. There were a total of 579 nucleotide positions in the final dataset. Phylogenetic analysis was conducted in MEGA5 (2). This analysis suggests as many as 7 clades of A. mellifera within Kenya. Whether this is evidence of more subspecies than previously described will require more sampling. Moreover, the weak statistical support suggests a need to expand beyond the ND2 region to describe East African subspecies of A. mellifera. Accession Numbers KJ628964-KJ628987. (DOCX) [file pone.0094459.s001.docx]

#
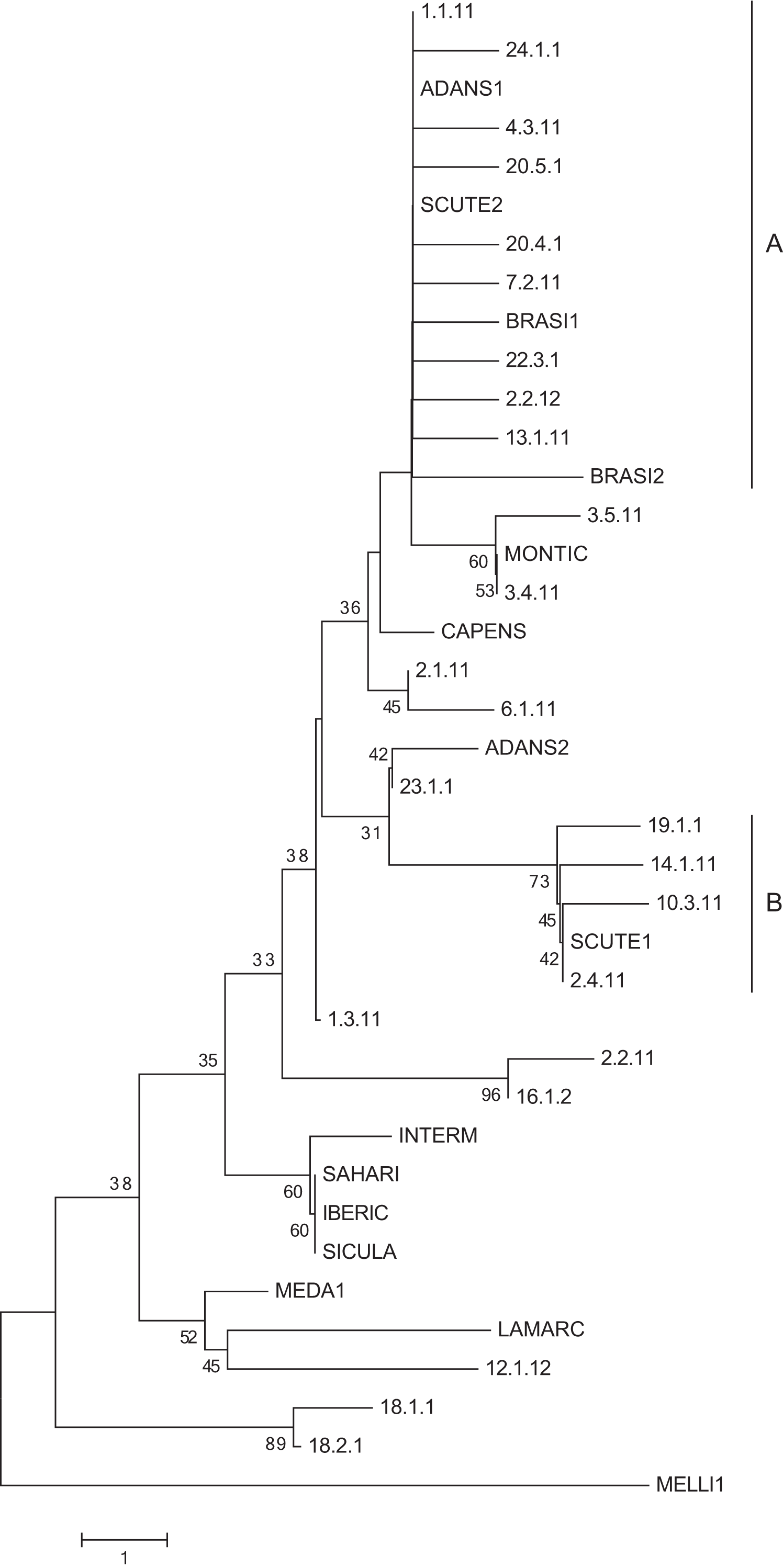


# Figure S1. Neighbor-joining tree (Saitou and Nei, 1987) comparing representative Kenyan honeybee haplotypes (see Table S2) with subspecies described in Arias and Sheppard 1996 (in capitals). The European subspecies *Apis mellifera mellifera* (MELLI1) is used as the outgroup. The percentage of replicate trees in which the associated taxa clustered together in the bootstrap test (2000 replicates) are shown next to the branches for values greater than 30% (1). Branch length indicates number of SNP differences. The analysis involved 39 nucleotide sequences from the ND2 mitochondrial region. Twenty-four of the 39 are representative unique haplotypes. For a list of all individuals represented by these haplotypes see Table S2. There were a total of 579 nucleotide positions in the final dataset. Phylogenetic analysis was conducted in MEGA5 (2). This analysis suggests as many as 7 clades of *A. mellifera* within Kenya. Whether this is evidence of more subspecies than previously described will require more sampling. Moreover, the weak statistical support suggests a need to expand beyond the ND2 region to describe East African subspecies of *A. mellifera*. Accession Numbers (to be added at publication).

1. Felsenstein, J (1985) Confidence limits on phylogenies: an approach utilizing the bootstrap. Evolution 39: 783–791.

2. Tamura K, Peterson D, Peterson N, Stecher G, Nei M, Kumar S (2011) **MEGA5: Molecular Evolutionary Genetics Analysis using maximum likelihood, evolutionary distance, and maximum parsimony methods.** Molecular Biology and Evolution 28:2731-2739.
