# Supplementary material for: Evaluation of the Distribution and Impacts of Parasites, Pathogens, and Pesticides on Honey Bee (Apis mellifera) Populations in East Africa
Source: PLoS One. 2014 Apr 16;9(4):e94459. doi: 10.1371/journal.pone.0094459 (PMC3989218; doi:10.1371/journal.pone.0094459)
Supplement: Figure S2 — Effect of location on colony weight and Varroa numbers. Colonies of from upland site 1 (icipe) were assayed in June 2010, immediately prior and after moving upland colonies from site 1 to site 25 on the coast. Coastal colonies were assayed at nearby apiaries (sites 26 and 27). There were no significant differences in colony weight (A, F(3,26 = 1.43), p = 0.256), but there were significantly fewer Varroa in colonies moved to site 25 (B, F(3,25) = 6.63, p = 0.0019). In August 2010, upland colonies at sites 1 and 25 and coastal colonies at site 25 were assayed. There were again no significant differences in weight (C, F(2, 16) = 1.29, p = 0.301), but both upland and coastal colonies had significantly fewer Varroa at site 25 (D, F(2,16) = 6.96, p = 0.0067). The number of colonies in each group at each timepoint is indicated at the bottom of each bar in B and D. Letters represent groups that were significantly different with a Tukey HSD post-hoc pairwise comparison, at p<0.05. While the graphs show the actual average numbers of Varroa, counts were converted to logarithmic scale for statistical analysis. (DOCX) [file pone.0094459.s002.docx]

**Effect of geographic location on Varroa levels and colony weight.**

In the 2010 survey, there was a significant positive association between *Varroa* levels and elevation. Elevation is correlated with landscape ecology and with subspecies (Ruttner 1987). In order to determine if environmental factors were contributing to colony *Varroa* levels, we moved upland colonies of *Apis mellifera* from the central region of Kenya (the *icipe* apiary in Nairobi, Kenya, site 1, 1602m elevation) to a coastal location (site 25, Bomani, -3°53.2450, 39°44.4600 41m).

Prior to the move (June 6-7, 2011), colonies at *icipe* were assayed for weight, number of frames of brood, and numbers of *Varroa*. After the move, site 1 colonies at site 25 and colonies at site 26 (Mtepeni, -3°55.0110, 39°44.6300, 23m) and site 27 (Kwentu, -3°56.8700, 39°42.7900, 10m) were assayed for the same parameters (June 13-15, 2011). Site 26 and 27 colonies were subsequently moved to site 25. In mid August 2011, colonies at site 1 and site 25 were re-assayed. Finally, in December 2011 and February 2012, any remaining colonies at site 25 were assayed. Prior to assaying the colonies, we confirmed that the original queen was still present in the colony. A one-way ANOVA followed by post-hoc Tukey HSD tests were used to determine if there were significant differences across the sample groups in June and August 2011, while a repeated measures ANOVA using genotype as a variable was used to determine if there were significant effects of genotype or time in the December 2011 and February 2012 samples. Analyses were performed using JMP 9.0.2 (SAS, Cary, NC). *Varroa* counts were logarithmically transformed for the statistical analyses.

Immediately after the relocation, there were no significant differences among the upland and coastal colonies in terms of weight (F(3,26)=1.43, p=0.256, Figure S2A) or brood area (F(3,25)=2.43, p=0.09, data not shown). Upland colonies moved to the coast however, had significantly lower *Varroa* levels than upland colonies at site 1 (F(3,25)=6.63, p=0.0019, Figure S2B). Two months after the relocation (August 2011), there were still no differences in colony weight (F(2, 16)=1.29, p=0.301, Figure S2C), but upland colonies at site 25 had significantly more brood than upland colonies still at site 1 (indigenous coastal colonies were intermediate; F(2,16)=4.03, p=0.04, data not shown) and upland colonies at site 25 and indigenous coastal colonies had significantly less *Varroa* than upland colonies at site 1 (F(2,16)=6.96, p=0.0067, Figure S2D). Evaluations of upland colonies at the coast and indigenous coastal colonies in subsequent months (December 2011 and February 2012) revealed no significant effect of genotype on weight (F(1,8)=0.0, p=1.0), *Varroa* levels (F(1,8)=0.26, p=0.62), or amount of brood (F(1,7)=0.17, p=0.69). *Varroa* levels did increase across the two timepoints (F(1,8)=7.17, p=0.03), but there was no significant effect of time on weight (F(1,8)=3.34, p=0.11) or amount of brood (F(1,7)=4.39, p=0.07).


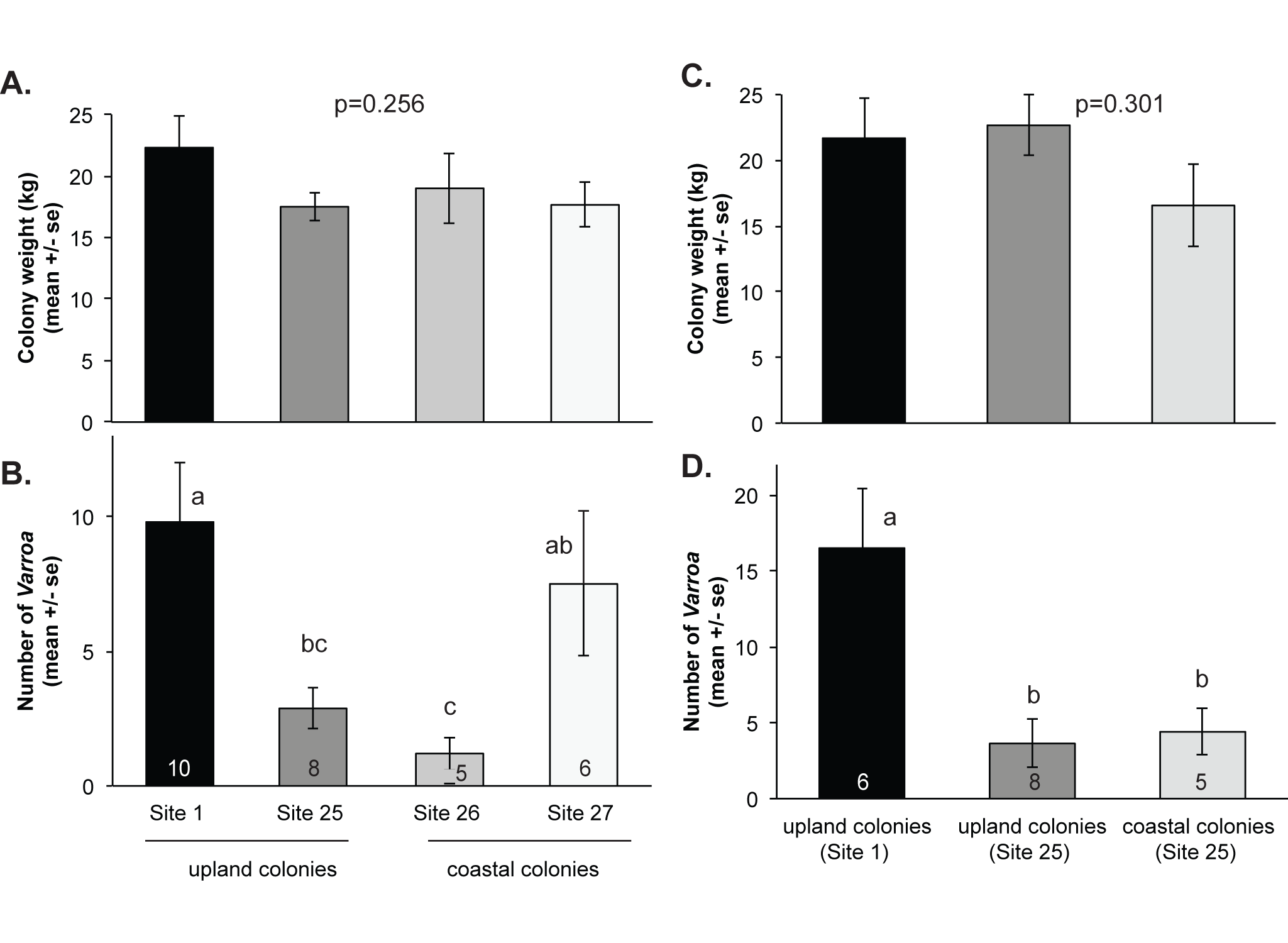


**Figure S2. Effect of location on colony weight and *Varroa* numbers.** Colonies of from upland site 1 (icipe) were assayed in June 2010, immediately prior and after moving upland colonies from site 1 to site 25 on the coast. Coastal colonies were assayed at nearby apiaries (sites 26 and 27). There were no significant differences in colony weight (**A**, F(3,26=1.43), p=0.256), but there were significantly fewer *Varroa* in colonies moved to site 25 (**B**, F(3,25)=6.63, p=0.0019). In August 2010, upland colonies at sites 1 and 25 and coastal colonies at site 25 were assayed. There were again no significant differences in weight (**C**, F(2, 16)=1.29, p=0.301), but both upland and coastal colonies had significantly fewer *Varroa* at site 25 (**D**, F(2,16)=6.96, p=0.0067). The number of colonies in each group at each timepoint is indicated at the bottom of each bar in B and D. Letters represent groups that were significantly different with a Tukey HSD post-hoc pairwise comparison, at p<0.05. While the graphs show the actual average numbers of *Varroa*, counts were converted to logarithmic scale for statistical analysis.

**References**

Ruttner F (1987) Biogeography and Taxonomy of Honeybees: Springer. 284 p.
