# Supplementary material for: Evaluation of the Distribution and Impacts of Parasites, Pathogens, and Pesticides on Honey Bee (Apis mellifera) Populations in East Africa
Source: PLoS One. 2014 Apr 16;9(4):e94459. doi: 10.1371/journal.pone.0094459 (PMC3989218; doi:10.1371/journal.pone.0094459)
Supplement: Table S1 — Apiary colony list indicating location, Varroa load, hygienic behavior, presence of virus, Nosema , pesticide and subspecies identification. Each apiary was given a site number and apiary name. Each colony has a unique identification number with the apiary number in the first position and the colony number in the second. Colony size is indicated by numbers of frames with bees. Total Varroa counts are based on standard sugar roll assay described in Ellis and Macedo, 2001 (1). The percent hygienic behavior (2) was calculated by taking the final number of fully and partially removed pupae/(207 – number originally uncapped or empty cells) *100. Boxes with UD are undetermined indicating measurements were not taken for these colonies. Positive for virus or Nosema apis detection per colony is indicated by an “X”. The presence of pesticide detected in wax or bee bread is indicated in parts per billion (ppb). Only four pesticides were detected: CP (chloropyrios), CT (chlorothalonil), N (1-naphtol), F (fluvalinate). ND2 subspecies identification is based on the analysis presented in Figure S1. Individuals from most of the colonies grouped with the scutellata A or B clades. Some were identical in sequence to A. mellifera monticola described by Arias and Sheppard, 1996 (3). Some individuals from colonies at sites 12, 13, 16 and 17 were most closely related to A. mellifera lamarckii and are thus called “lamarckii-like”. Colonies at site 18 were unique and perhaps represent a distinct subspecies (A. m. simensis or yemenitica). A few individuals did not group with any previously described subspecies and are thus indicated as UD. Interestingly three colonies (2.1, 2.2, and 16.1) had individuals from multiple mitochondrial lineages. (DOCX) [file pone.0094459.s003.docx]

| **Site** | **Site Name** | **Colony ID** | **Coordinates** | **Elevation** | **# of Frames**  **with Adult Bees** | **Total Varroa** | **Hygienic**  **Behavior** | **BQCV** | **DWV** | **ABPV** | **Wax (ppb)** | **Bee**  **Bread (ppb)** | **N. apis** | **ND2 subspecies** |
| --- | --- | --- | --- | --- | --- | --- | --- | --- | --- | --- | --- | --- | --- | --- |
| 1 | Kasarani (ICIPE) | 1.1 | -1°13.3631, 36°53.7867 | 1602m | 9.5 | 9 | 71.5 |  |  |  | CP(4.0) | CT(31.0); CP(6.9) |  | scutellata A |
|  |  | 1.2 |  |  | 8 | 21 |  |  | ✕ |  |  |  |  | scutellata A |
|  |  | 1.3 |  |  | 9 | 21 | 42 |  | ✕ |  |  |  |  | UD |
|  |  | 1.4 |  |  | 4 | 10 | 35 |  |  |  |  |  |  | scutellata A |
|  |  | 1.5 |  |  | 12 | 28 | 0 |  |  |  |  |  |  | UD |
| 2 | SEUCO (Kitui) | 2.1 | -1°18.3005, 37°45.9075 | 1150m | 6 | NA |  |  |  |  |  |  |  | scutellata B, UD |
|  |  | 2.2 |  |  |  | NA |  |  | ✕ |  |  |  |  | scutellata A, UD |
|  |  | 2.3 |  |  | 8 | NA |  | ✕ |  |  |  |  |  | scutellata A |
|  |  | 2.4 |  |  | 8 | NA |  |  |  |  |  |  |  | scutellata B |
|  |  | 2.5 |  |  | 10 | NA |  | ✕ |  |  |  |  |  | scutellata B |
| 3 | Marchorwe | 3.1 | -0°30.0499, 36°32.7342 | 2509m | 8 | 57 | 19 | ✕ |  |  | CP(3.8) |  |  | scutellata A |
|  |  | 3.2 |  |  | 3 | 4 | 55 | ✕ |  |  |  |  |  | scutellata A |
|  |  | 3.3 |  |  | 5 | 71 | 35 | ✕ |  |  |  |  |  | scutellata B |
|  |  | 3.4 |  |  | 8 | 61 | 31 | ✕ |  |  |  |  |  | monticola |
|  |  | 3.5 |  |  | 2 | 1 |  | ✕ |  |  |  |  |  | monticola |
| 4 | Malewa | 4.1 | -0°31.5024, 36°24.1969 | 1973m | 10 | 11 | 21.8 |  | ✕ |  |  | CT(31.0); CP(1.9) |  | scutellata B |
|  |  | 4.2 |  |  | 9 | 14 | 40 |  |  |  |  |  |  | scutellata A |
|  |  | 4.3 |  |  | 10 | 46 | 1.9 |  | ✕ |  |  |  |  | scutellata A |
|  |  | 4.4 |  |  | 12 | 8 | 13.8 |  | ✕ |  |  |  |  | scutellata A |
|  |  | 4.5 |  |  | 9 | 5 | 94 |  |  |  |  |  |  | scutellata B |
| 5 | Ngeta | 5.1 | -0°34.6936, 36°37.7921 | 2627m | 4.5 | 2 |  | ✕ |  |  |  | CT(31.7); CP(33.6) |  | scutellata A |
| 6 | Nadasa | 6.1 | -0°31.2173, 36°37.3477 | 2549m | 5 | 5 |  | ✕ |  |  | CT(31.7) | CT(31.4) |  | UD |
|  |  | 6.2 |  |  | 6 | 13 |  | ✕ |  |  |  |  |  | scutellata A |
| 7 | Upper Kamuieti 1 | 7.1 | -0°21.1484, 37°18.9650 | 2233m |  | 22 |  | ✕ |  |  | CT(31.7) |  |  | scutellata A |
|  |  | 7.2 |  |  |  | 34 |  | ✕ |  |  |  |  |  | scutellata A |
| 8 | Upper Kamuieti 2 | 8.1 | -0°21.5432, 37°18.7934 | 2128m |  | 48 |  | ✕ |  |  | CT(26.7) | CT(31.2) |  | scutellata A |
| 9 | Nji-ini forest | 9.1 | -0°31.1364, 37°25.2342 | 1580m | 5 | 11 |  | ✕ | ✕ |  | N(116) | CT(31.5); CP(2.7) |  | scutellata B |
|  |  | 9.2 |  |  | 10 | 10 |  | ✕ | ✕ |  |  |  |  | scutellata A |
| 10 | Ichiara | 10.1 | -0°28.0380, 37°43.0370 | 436m | 7 | 18 |  | ✕ |  |  | CT(31.9) |  |  | scutellata B |
|  |  | 10.2 |  |  | 6 | 4 |  | ✕ |  |  |  |  |  | scutellata B |
|  |  | 10.3 |  |  | 6 | 16 |  | ✕ |  |  |  |  |  | scutellata B |
| 11 | Taita Hills | 11.1 | -3°28.6817, 38°20.4598 | 1591m | 6 | 0 | 95.7 | ✕ |  |  | CT(31.2) | CT(39.9); F(13.3) |  | scutellata B |
|  |  | 11.2 |  |  | 5 | 6 | 27.7 | ✕ |  |  |  |  |  | scutellata B |
|  |  | 11.3 |  |  | 5 | 0 |  |  |  |  |  |  |  | scutellata B |
|  |  | 11.4 |  |  | 2.5 | 9 |  |  |  |  |  |  |  | scutellata B |
|  |  | 11.5 |  |  | 2 | 0 |  | ✕ |  |  |  |  |  | scutellata B |
| 12 | Gete Ruins | 12.1 | -3°18.3899, 40°1.0793 | 36m | 10 | 8 | 8 | ✕ |  |  | CT(59.8); F(18.9) | CT(31.0) | ✕ | lamarckii-like |
|  |  | 12.2 |  |  | 9 | 6 |  | ✕ | ✕ |  |  |  | ✕ | lamarckii-like |
|  |  | 12.3 |  | 36m | 10 | 1 | 33.5 | ✕ |  |  |  |  | ✕ | scutellata B |
|  |  | 12.4 |  |  | 5 | 4 | 93 | ✕ | ✕ |  |  |  |  | lamarckii-like |
|  |  | 12.5 |  |  | 8 | 67 |  | ✕ | ✕ |  |  | CT(31.2) | ✕ | scutellata A |
| 13 | Oceanside | 13.1 | -3°20.2514, 39°59.1495 | 15m | 10 | 0 | 63 |  |  |  |  |  | ✕ | scutellata A |
|  |  | 13.2 |  |  | 10 | 2 |  | ✕ |  |  |  |  | ✕ | scutellata B |
|  |  | 13.3 |  |  | 11 | 2 | 30 |  | ✕ |  |  |  | ✕ | lamarckii-like |
|  |  | 13.4 |  |  | 10 | 5 | 59.5 |  | ✕ | ✕ |  |  | ✕ | scutellata B |
|  |  | 13.5 |  |  | 13 | 1 | 81.6 |  |  |  |  |  | ✕ | scutellata B |
| 14 | South Coast (Kaya Mukawa) | 14.1 | -4°19.7239, 39°31.0820 | 68m | 10 | 14 | 0 | ✕ | ✕ |  | CT (31.4) | CT(31.0) |  | scutellata B |
|  |  | 14.2 |  |  | 4 | 6 | 94.8 | ✕ | ✕ |  |  |  |  | scutellata B |
| 15 | Tanzania Border | 15.1 | -4°31.7394, 39°9.2171 | 62m | 2.5 | 0 | 78 |  |  |  | CT(30.9) | CT(30.9) |  | scutellata B |
|  |  | 15.2 |  |  | 3 | 0 | 98.3 |  |  |  |  |  |  | scutellata B |
|  |  | 15.3 |  |  | 2 | 0 |  |  |  |  |  |  | ✕ | scutellata B |
|  |  | 15.4 |  |  | 5 | 6 |  |  |  |  |  |  |  | scutellata B |
| 16 | Mandera Town 1 | 16.1 | 3°56.2050, 41°52.0900 | 212m |  | NA |  |  |  |  |  |  |  | scutellata A, lamarckii-like, UD |
| 17 | Mandera Town 2 | 17.1 | 3°56.1970, 41°52.0820 | 221m |  | 0 |  |  |  |  |  |  |  | lamarckii-like |
| 18 | Mandera West | 18.1 | 3°53.3790, 40°16.0440 | 894m |  | 0 |  |  |  |  |  |  |  | simensis or yemenitica |
|  |  | 18.2 |  |  |  | 0 |  |  |  |  |  |  |  | simensis or yemenitica |
|  |  | 18.3 |  |  |  | 0 |  |  |  |  |  |  |  | scutellata A |
| 19 | Mt. Elgon, Sasuri | 19.1 | 0°50.1030, 34°28.5060 | 2006m |  | NA |  | ✕ |  |  |  |  |  | scutellata B |
|  |  | 19.2 |  |  |  | NA |  | ✕ |  |  |  |  |  | scutellata A |
|  |  | 19.3 |  |  |  | NA |  | ✕ | ✕ |  |  |  |  | scutellata A |
| 20 | Mt. Elgon, Kareu | 20.1 | 0°48.0780, 34°27.1580 | 1543m | 7 | 20 | 48.6 | ✕ | ✕ |  |  |  |  | scutellata A |
|  |  | 20.2 |  |  | 6 | 9 | 18.7 | ✕ |  |  |  |  |  | scutellata A |
|  |  | 20.3 |  |  | 4 | 8 | 46.0 | ✕ | ✕ |  |  |  |  | scutellata A |
|  |  | 20.4 |  |  | 9 | 30 | 77.7 | ✕ | ✕ |  |  |  |  | scutellata A |
|  |  | 20.5 |  |  | 10 | 25 | 100.0 | ✕ | ✕ |  |  |  |  | scutellata A |
| 21 | Mt. Elgon, Chepkui | 21.1 | 0°49.543, 34°42.1740 | 1869m | 9 | 38 | 34.9 | ✕ | ✕ |  |  |  |  | scutellata A |
|  |  | 21.2 |  |  | 10 | 49 | 22.5 | ✕ | ✕ |  |  |  |  | scutellata B |
|  |  | 21.3 |  |  | 12 | 20 | 100.0 | ✕ | ✕ |  |  |  |  | scutellata A |
|  |  | 21.4 |  |  | 5 | 10 | 97.3 | ✕ |  |  |  |  |  | UD |
|  |  | 21.5 |  |  | 14 | 12 | 81.5 | ✕ | ✕ |  |  |  |  | monticola |
| 22 | Mt. Elgon, Moorland | 22.1 | 0°57.2460, 34°36.2860 | 2956m |  | NA | NA | ✕ |  |  |  |  | ✕ | monticola |
|  |  | 22.2 |  |  |  | NA | NA |  |  |  |  |  | ✕ | monticola |
|  |  | 22.3 |  |  |  | NA | NA |  |  |  |  |  |  | scutellata A |
| 23 | Busende Village | 23.1 | 0°22.2330, 34°9.2920 | 1164m |  | 11 | NA |  | ✕ |  |  |  |  | UD |
|  |  | 23.2 |  |  |  | 7 | NA | ✕ | ✕ |  |  |  |  | UD |
|  |  | 23.3 |  |  |  | 6 | NA | ✕ |  |  |  |  |  | scutellata A |
|  |  | 23.4 |  |  |  | 8 | NA | ✕ |  |  |  |  |  | scutellata B |
|  |  | 23.5 |  |  |  | 2 | NA | ✕ | ✕ |  |  |  |  | scutellata A |
| 24 | Lugala | 24.1 | 0°19.2430, 34°5.5000 | 1190m |  | NA | NA | ✕ | ✕ |  |  |  |  | scutellata A |
|  |  | 24.2 |  |  |  | NA | NA |  | ✕ |  |  |  |  | scutellata A |
|  |  | 24.3 |  |  |  | NA | NA | ✕ |  |  |  |  |  | scutellata A |
| 25 | Bomani |  | -3°53.2450, 39°44.4600 | 41m |  |  |  |  |  |  |  |  |  |  |
| 26 | Mtepeni |  | -3°55.0110, 39°44.6300 | 23m |  |  |  |  |  |  |  |  |  |  |
| 27 | Kwetu |  | -3°56.8700, 39°42.7900 | 10m |  |  |  |  |  |  |  |  |  |  |

**Table S1.** **Apiary colony list indicating location, *Varroa* load, hygienic behavior, presence of virus, *Nosema,* pesticide and subspecies identification.** Each apiary was given a site number and apiary name. Each colony has a unique identification number with the apiary number in the first position and the colony number in the second. Colony size is indicated by numbers of frames with bees. Total *Varroa* counts are based on standard sugar roll assay described in Ellis and Macedo, 2001 (1). The percent hygienic behavior (2) was calculated by taking the final number of fully and partially removed pupae/(207 – number originally uncapped or empty cells) *100. Boxes with UD are undetermined indicating measurements were not taken for these colonies. Positive for virus or *Nosema apis* detection per colony is indicated by an "X". The presence of pesticide detected in wax or bee bread is indicated in parts per billion (ppb). Only four pesticides were detected: CP (chloropyrios), CT (chlorothalonil), N (1-naphtol), F (fluvalinate). ND2 subspecies identification is based on the analysis presented in Figure S1. Individuals from most of the colonies grouped with the scutellata A or B clades. Some were identical in sequence to *A. mellifera monticola* described by Arias and Sheppard, 1996 (3). Some individuals from colonies at sites 12, 13, 16 and 17 were most closely related to *A. mellifera lamarckii* and are thus called "lamarckii-like". Colonies at site 18 were unique and perhaps represent a distinct subspecies (*A. m.* *simensis* or *yemenitica*). A few individuals did not group with any previously described subspecies and are thus indicated as UD. Interestingly three colonies (2.1, 2.2, and 16.1) had individuals from multiple mitochondrial lineages.

1. Ellis MD, Macedo PA (2001) G01-1430 Using the Sugar Roll Technique to Detect Varroa Mites in Honey Bee Colonies. Extension. Lincoln, NE, University of Nebraska: 4.

2. Spivak M, Reuter GS (1998) Honey bee hygienic behavior. American Bee Journal 138: 283-286.

3. Arias MC, Sheppard WS (1996) Molecular phylogenetics of honey bee subspecies (Apis mellifera L.) inferred from mitochondrial DNA sequence." Molecular Phylogenetics and Evolution 5: 557-66.
