# Supplementary material for: Evaluation of the Distribution and Impacts of Parasites, Pathogens, and Pesticides on Honey Bee (Apis mellifera) Populations in East Africa
Source: PLoS One. 2014 Apr 16;9(4):e94459. doi: 10.1371/journal.pone.0094459 (PMC3989218; doi:10.1371/journal.pone.0094459)
Supplement: Table S2 — Type of data collected at each apiary. An “X” indicates the data type at the top of the column was collected in at least one colony in the corresponding apiary. See Materials and Methods for a full description of the type of data collected and the analyses that were performed. (DOCX) [file pone.0094459.s004.docx]

**Table S2.** **Type of data collected at each apiary.** An "X" indicates the data type at the top of the column was collected in at least one colony in the corresponding apiary. See Materials and Methods for a full description of the type of data collected and the analyses that were performed.

| **Site** | **Site Name** | **Varroa**  **mites** | **Nosema**  **spp.** | **Viruses** | **Hygienic**  **Behavior** | **Population**  **Survey** | **ND2 subspecies** | **Pesticide** |
| --- | --- | --- | --- | --- | --- | --- | --- | --- |
| 1 | Kasarani (ICIPE) | ✕ | ✕ | ✕ | ✕ | ✕ | ✕ | ✕ |
| 2 | SEUCO (Kitui) |  |  | ✕ |  | ✕ | ✕ | ✕ |
| 3 | Marchorwe | ✕ | ✕ | ✕ | ✕ | ✕ | ✕ | ✕ |
| 4 | Malewa | ✕ | ✕ | ✕ | ✕ | ✕ | ✕ | ✕ |
| 5 | Ngeta | ✕ |  | ✕ |  | ✕ | ✕ | ✕ |
| 6 | Nadasa | ✕ |  | ✕ |  | ✕ | ✕ | ✕ |
| 7 | Upper Kamuieti 1 | ✕ |  | ✕ |  | ✕ | ✕ | ✕ |
| 8 | Upper Kamuieti 2 | ✕ |  | ✕ |  |  | ✕ | ✕ |
| 9 | Nji-ini forest | ✕ |  | ✕ |  | ✕ | ✕ | ✕ |
| 10 | Ichiara | ✕ | ✕ | ✕ |  | ✕ | ✕ | ✕ |
| 11 | Taita Hills | ✕ | ✕ | ✕ | ✕ | ✕ | ✕ | ✕ |
| 12 | Gete Ruins | ✕ | ✕ | ✕ | ✕ | ✕ | ✕ | ✕ |
| 13 | Oceanside | ✕ | ✕ | ✕ | ✕ | ✕ | ✕ | ✕ |
| 14 | South Coast (Kaya Mukawa) | ✕ | ✕ | ✕ | ✕ | ✕ | ✕ | ✕ |
| 15 | Tanzania Border | ✕ | ✕ | ✕ | ✕ | ✕ | ✕ | ✕ |
| 16 | Mandera Town 1 |  | ✕ | ✕ |  |  | ✕ |  |
| 17 | Mandera Town 2 | ✕ | ✕ | ✕ |  |  | ✕ |  |
| 18 | Mandera West | ✕ | ✕ | ✕ |  |  | ✕ |  |
| 19 | Mt. Elgon, Sasuri |  |  | ✕ |  |  | ✕ |  |
| 20 | Mt. Elgon, Kareu | ✕ | ✕ | ✕ | ✕ |  | ✕ |  |
| 21 | Mt. Elgon, Chepkui | ✕ | ✕ | ✕ | ✕ |  | ✕ |  |
| 22 | Mt. Elgon, Moorland |  | ✕ | ✕ |  |  | ✕ |  |
| 23 | Busende Village | ✕ | ✕ | ✕ |  |  | ✕ |  |
| 24 | Lugala |  |  | ✕ |  |  | ✕ |  |
