# Supplementary material for: Evaluation of the Distribution and Impacts of Parasites, Pathogens, and Pesticides on Honey Bee (Apis mellifera) Populations in East Africa
Source: PLoS One. 2014 Apr 16;9(4):e94459. doi: 10.1371/journal.pone.0094459 (PMC3989218; doi:10.1371/journal.pone.0094459)
Supplement: Table S3 — A list of ND2 haplotypes used for comparison. Individuals were sequenced for the ND2 region (as described in 1) from 24 apiaries across Kenya. There were 24 unique haplotypes from 109 individuals sequenced. Each column is headed by the representative haplotype (in bold) used for analysis. Haplotypes identical to the representative type follow in each column. The columns are the far right is a list of unique single haplotypes. Numeric designations for each individual follow the following scheme: apiary.colony.individual. (DOCX) [file pone.0094459.s005.docx]

| **1.1.11** | **1.3.11** | **2.2.11** | **2.2.12** | **2.4.11** | **3.4.11** | **6.1.11** | **12.1.12** | **18.1.1** | **19.1.1** | **2.1.11** |
| --- | --- | --- | --- | --- | --- | --- | --- | --- | --- | --- |
| 1.2.11 | 1.5.11 | 9.2.11 | 2.3.11 | 2.1.12 | 3.4.12 | 21.4.1 | 12.1.11 | 18.1.3 | 19.1.2 | **3.5.11** |
| 1.4.11 | 23.2.1 |  | 2.2.15 | 2.5.11 | 21.5.1 |  | 12.2.11 | 18.1.5 |  | **4.3.11** |
| 1.4.15 |  |  | 16.1.3 | 3.3.11 | 22.1.2 |  | 12.2.12 |  |  | **7.2.11** |
| 3.1.11 |  |  | 16.1.4 | 4.1.11 | 22.1.3 |  | 12.2.15 |  |  | **10.3.11** |
| 3.2.11 |  |  | 16.1.5 | 4.5.12 | 22.2.1 |  | 12.4.11 |  |  | **13.1.11** |
| 4.2.12 |  |  | 18.3.1 | 9.1.11 | 22.2.2 |  | 13.3.11 |  |  | **14.1.11** |
| 4.2.15 |  |  |  | 10.1.11 | 22.2.5 |  | 16.1.11 |  |  | **16.1.2** |
| 4.4.11 |  |  |  | 10.2.11 |  |  | 17.1.1 |  |  | **18.2.1** |
| 5.1.11 |  |  |  | 11.1.11 |  |  | 17.1.2 |  |  | **20.4.1** |
| 5.1.12 |  |  |  | 11.2.11 |  |  | 17.1.3 |  |  | **20.5.1** |
| 5.1.13 |  |  |  | 11.3.11 |  |  | 17.1.4 |  |  | **23.1.1** |
| 6.2.11 |  |  |  | 11.4.11 |  |  | 17.1.5 |  |  | **22.3.1** |
| 7.1.12 |  |  |  | 11.5.11 |  |  |  |  |  | **24.1.1** |
| 8.1.11 |  |  |  | 12.3.11 |  |  |  |  |  |  |
| 8.1.13 |  |  |  | 13.2.11 |  |  |  |  |  |  |
| 12.5.11 |  |  |  | 13.4.11 |  |  |  |  |  |  |
| 19.2.1 |  |  |  | 13.4.15 |  |  |  |  |  |  |
| 19.2.3 |  |  |  | 13.5.11 |  |  |  |  |  |  |
| 19.3.1 |  |  |  | 14.2.11 |  |  |  |  |  |  |
| 20.1.1 |  |  |  | 14.2.12 |  |  |  |  |  |  |
| 20.2.1 |  |  |  | 15.2.11 |  |  |  |  |  |  |
| 20.3.1 |  |  |  | 15.3.11 |  |  |  |  |  |  |
| 21.1.2 |  |  |  | 15.4.11 |  |  |  |  |  |  |
| 21.3.1 |  |  |  | 21.2.1 |  |  |  |  |  |  |
| 21.3.15 |  |  |  | 23.4.1 |  |  |  |  |  |  |
| 23.3.1 |  |  |  |  |  |  |  |  |  |  |
| 23.5.1 |  |  |  |  |  |  |  |  |  |  |
| 24.2.1 |  |  |  |  |  |  |  |  |  |  |
| 24.3.1 |  |  |  |  |  |  |  |  |  |  |

**Table S3.** **A list of ND2 haplotypes used for comparison.** Individuals were sequenced for the ND2 region (as described in 1) from 24 apiaries across Kenya. There were 24 unique haplotypes from 109 individuals sequenced. Each column is headed by the representative haplotype (in bold) used for analysis. Haplotypes identical to the representative type follow in each column. The columns are the far right is a list of unique single haplotypes. Numeric designations for each individual follow the following scheme: apiary.colony.individual.

1. Arias MC, Sheppard WS (1996) Molecular phylogenetics of honey bee subspecies (Apis mellifera L.) inferred from mitochondrial DNA sequence." Molecular Phylogenetics and Evolution 5: 557-66.
