# Supplementary material for: Evaluation of the Distribution and Impacts of Parasites, Pathogens, and Pesticides on Honey Bee (Apis mellifera) Populations in East Africa
Source: PLoS One. 2014 Apr 16;9(4):e94459. doi: 10.1371/journal.pone.0094459 (PMC3989218; doi:10.1371/journal.pone.0094459)
Supplement: Table S4 — ND2 region variable sites from Kenya honeybee haplotypes compared to the subspecies A. m. adansonii (ADANS2) (1). The nucleotide positions starting from the ND2 ATC (isoleucine) are indicated in the top row with corresponding position numbers from the complete honeybee mitochondrial genome (2). The codon position for each SNP is indicated at the bottom of the figure. Over the 579 bp of the ND2 coding region 85% of SNPs were in the third codon position. Three SNPs were in the second codon position. The first is a transversion at position 53 (T↔A) that results in an amino acid change of isoleucine ↔ asparagine. A second codon position change is a transition at nucleotide position 161 (C↔T) resulting in an amino acid change of threonine ↔ isoleucine. The third second codon change (position 458) is also a transition (C↔T) resulting in threonine ↔ isoleucine. The Kenya honeybee population also shows a first codon transition (position 412; G↔A) that results in an amino acid difference (valine ↔ isoleucine) when compared to the reference sequence. (DOCX) [file pone.0094459.s006.docx]

**Table S4.** **ND2 region variable sites from Kenya honeybee haplotypes compared to the subspecies *A. m. adansonii* (ADANS2)** (1). The nucleotide positions starting from the ND2 ATC (isoleucine) are indicated in the top row with corresponding position numbers from the complete honeybee mitochondrial genome (2). The codon position for each SNP is indicated at the bottom of the figure. Over the 579 bp of the ND2 coding region 85% of SNPs were in the third codon position. Three SNPs were in the second codon position. The first is a transversion at position 53 (T ↔ A) that results in an amino acid change of isoleucine ↔ asparagine. A second codon position change is a transition at nucleotide position 161 (C ↔ T) resulting in an amino acid change of threonine ↔ isoleucine. The third second codon change (position 458) is also a transition (C ↔ T) resulting in threonine ↔ isoleucine. The Kenya honeybee population also shows a first codon transition (position 412; G ↔ A) that results in an amino acid difference (valine ↔ isoleucine) when compared to the reference sequence.

| From ATC | 9 | 36 | 51 | 53 | 54 | 57 | 84 | 150 | 156 | 161 | 162 | 165 | 240 | 270 | 312 | 321 | 336 | 366 | 387 | 399 | 412 | 456 | 458 | 498 | 516 | 552 | 576 |
| --- | --- | --- | --- | --- | --- | --- | --- | --- | --- | --- | --- | --- | --- | --- | --- | --- | --- | --- | --- | --- | --- | --- | --- | --- | --- | --- | --- |
| Mito | 511 | 538 | 553 | 555 | 556 | 559 | 586 | 652 | 658 | 663 | 664 | 667 | 742 | 772 | 814 | 823 | 838 | 868 | 889 | 901 | 914 | 958 | 960 | 1000 | 1018 | 1054 | 1078 |
| ADANS1 | C | C | T | T | C | T | T | C | T | C | C | G | T | T | C | T | T | T | C | A | G | T | C | C | A | T | T |
| 1.1.11 |  |  |  |  |  |  |  |  |  |  |  |  |  |  |  |  |  |  |  |  |  |  |  |  |  |  |  |
| 1.3.11 |  |  |  |  |  |  |  |  |  |  |  |  |  |  |  |  |  |  | T |  |  |  |  |  |  |  |  |
| 2.1.11 |  |  |  |  |  |  |  |  |  |  |  | A |  |  |  |  |  |  |  |  |  |  |  |  |  |  |  |
| 2.2.11 |  | T | A |  | T |  | C |  |  |  |  |  |  |  |  |  |  |  | T |  |  |  |  |  |  |  |  |
| 2.2.12 |  |  |  |  |  |  |  |  |  |  |  |  |  |  |  |  |  |  |  |  |  |  |  |  |  | C |  |
| 2.4.11 |  |  |  |  |  |  |  |  | C |  |  | A |  |  |  |  |  |  | T |  |  |  |  |  |  |  | C |
| 3.4.11 |  |  |  |  |  |  |  |  |  |  |  |  |  |  |  |  |  |  |  |  |  |  |  |  | T |  |  |
| 3.5.11 |  |  |  |  |  |  |  |  |  |  |  |  |  |  |  |  |  |  |  |  | A |  |  |  | T |  |  |
| 4.3.11 |  |  |  |  |  |  |  |  |  |  |  |  |  | C |  |  |  |  |  |  |  |  |  |  |  |  |  |
| 6.1.11 |  |  |  |  |  |  |  |  |  |  |  | A |  |  |  |  |  |  |  | T |  |  |  |  |  |  |  |
| 7.2.11 |  |  |  |  |  |  |  |  |  |  |  |  |  |  |  |  |  | C |  |  |  |  |  |  |  |  |  |
| 10.3.11 |  |  |  |  |  |  |  |  | C |  |  | A |  |  |  |  |  |  | T |  |  |  | T |  |  |  | C |
| 12.1.12 |  |  |  |  |  |  |  | T |  |  | T |  | C |  |  | C |  |  | T |  |  | C |  | T |  |  |  |
| 13.1.11 |  |  |  |  |  |  |  |  |  |  |  |  |  |  | T |  |  |  |  |  |  |  |  |  |  |  |  |
| 14.1.11 |  |  |  |  |  |  |  |  | C |  |  | A |  |  | T |  |  |  | T |  |  |  |  |  |  |  | C |
| 16.1.2 |  | T |  |  | T |  | C |  |  |  |  |  |  |  |  |  |  |  | T |  |  |  |  |  |  |  |  |
| 18.1.1 |  | T |  |  | T | C |  |  |  |  | T |  | C |  |  |  |  |  | T |  |  |  |  | T |  | C |  |
| 18.2.1 |  |  |  |  | T | C |  |  |  |  | T |  | C |  |  |  |  |  | T |  |  |  |  | T |  | C |  |
| 19.1.1 |  |  |  |  |  |  |  |  | C |  |  | A |  |  |  |  |  |  | T |  | A |  |  |  |  |  | C |
| 20.4.1 |  |  |  | A |  |  |  |  |  |  |  |  |  |  |  |  |  |  |  |  |  |  |  |  |  |  |  |
| 20.5.1 |  |  |  |  |  |  |  |  |  |  |  |  |  |  |  |  | C |  |  |  |  |  |  |  |  |  |  |
| 23.1.1 |  |  |  |  |  |  |  |  |  |  |  | A |  |  |  |  |  |  | T |  |  |  |  |  |  |  |  |
| 22.3.1 | T |  |  |  |  |  |  |  |  |  |  |  |  |  |  |  |  |  |  |  |  |  |  |  |  |  |  |
| 24.1.1 |  |  |  |  |  |  |  |  |  | T |  |  |  |  |  |  |  |  |  |  |  |  |  |  |  |  |  |
| Codon | 3 | 3 | 3 | 2 | 3 | 3 | 3 | 3 | 3 | 2 | 3 | 3 | 3 | 3 | 3 | 3 | 3 | 3 | 3 | 3 | 1 | 3 | 2 | 3 | 3 | 3 | 3 |

1. Arias MC, Sheppard WS (1996) Molecular phylogenetics of honey bee subspecies (*Apis mellifera* L.) inferred from mitochondrial DNA sequence." Molecular Phylogenetics and Evolution 5: 557-66.

2. Crozier RH, Crozier YC (1993) The mitochondrial genome of the honeybee *Apis mellifera:* complete sequence and the genome organization. *Genetics* 133**:** 97–117.
