# Supplementary material for: Evaluation of the Distribution and Impacts of Parasites, Pathogens, and Pesticides on Honey Bee (Apis mellifera) Populations in East Africa
Source: PLoS One. 2014 Apr 16;9(4):e94459. doi: 10.1371/journal.pone.0094459 (PMC3989218; doi:10.1371/journal.pone.0094459)
Supplement: Form S1 — Collaborating authories. (DOCX) [file pone.0094459.s007.docx]

**Form S1. Collaborating authories.**

All samples were collected at maintained apiaries and consent was given by the collaborating authority. In all cases the owner, in the case of private land, or relevant authority, in the case of public land, gave permission for collections. See the Supplementary Materials for a list of the apiaries and owners. The following list indicates site; private or public, authority, and position. (1, Kasarani (ICIPE); private; Christian Borgemeister, Director General), (2, SEUCO (Kitui); Elliud Muli; professor, apiarist), (3, Muchorwe; private; Richard Karwigi ; apiarist and group chairman), (4, Malewa; private; Francis Matheri; apiarist), (5, Ngeta; public land private apiarist with permission; Macharia Wagithomo; apiarist), (6, Nadasa; private; Peter Wanjohi; apiarist), (7, Upper Kamuieti 1; public land private apiarist with permission; Ndegwa Njiru; apiarist), (8, Upper Kamuieti 2; public land private apiarist with permission; Ndegwa Njiru; apiarist), (9, Nji-ini forest; public; Paul Kiambo Karwigi; apiarist), (10, Ichiara; private; Elias Munyi; apiarist), (11, Taita Hills; public; Angliton Mwanjeve; apiarist), (12, Gete Ruins; public; Benson Tsuma; apiarist), (13, Oceanside; private; Joyce Mramba; apiarist), (14, South Coast (Kaya Muhaka); private; Saidi Suleiman; apiarist), (15, Tanzania Border; private; Elija Hodata Brunto; apiarist), (16, Mandera Town 1; private; Mohamoud Duale; apiarist), (17, Mandera Town 2; private; Mohamoud Duale; apiarist), (18, Mandera West; private; Abdi Osman; apiarist), (19, Mt. Elgon, Sasuri; private; M. Masake; apiarist), (20, Mt. Elgon, Kareu; private; Morris Kimnyanja; apiarist), (21; Mt. Elgon, Chepkui; private; M. Masake; apiarist), (22, Mt. Elgon, Moorland; private; M. Gamaliel Osikat; apiarist), (23, Busende Village; private; M. Masake; apiarist), (24, Lugala; private; M. Masake; apiarist), (25, Bomani; private; Benson Tsuma; apiarist), (26, Mtepeni; private; Benson Tsuma; apiarist), (27, Kwetu; private; Benson Tsuma; apiarist).
